# Distinct Foliar Uptake Pathways for Phosphorus and Nano‐Hydroxyapatite in Potato Revealed By Synchrotron μCT and ³³P Imaging

**DOI:** 10.1111/pce.70558

**Published:** 2026-04-22

**Authors:** Max Frank, Stine Le Tougaard, Augusta Szameitat, Emil Visby Kristensen, Daniel P. Persson, Rajmund Mokso, Søren Husted

**Affiliations:** ^1^ Department for Plant and Environmental Sciences (PLEN) Copenhagen University, Faculty of Science Frederiksberg Denmark; ^2^ Department of Physics Technical University of Denmark (DTU) Lyngby Denmark

**Keywords:** bioimaging, foliar fertilisation, laser ablation ICP‐MS, micro computed tomography, nanoparticles, phosphor screen imaging, phosphorus nutrition, potato, *Solanum tuberosum*, uptake efficiency

## Abstract

Potato (*Solanum tuberosum* L.) has a high phosphorus (P) requirement, yet its shallow root system and the strong P‐fixing capacity of many soils limit the effectiveness of soil fertilisation. Foliar application of nano‐hydroxyapatite particles (nHAPs) has emerged as a promising alternative P fertilisation practice. In this study, we compared foliar uptake mechanisms of nHAPs and orthophosphate ions (Pi) in potato using advanced bioimaging techniques and quantified their P uptake efficiency (PUE) with ³³P radioisotopes in controlled‐environment and field experiments. Imaging revealed distinct uptake pathways: Pi penetrated directly through the cuticle, whereas nHAPs primarily entered through hydraulically activated stomata. Foliar application of nHAPs achieved a PUE of approximately 50% after 7 days ‐ far exceeding global averages for soil‐applied P fertilisers ‐ while avoiding the leaf scorching commonly associated with Pi sprays. Pi uptake was insensitive to leaf surface polarity but increased when the surface tension of the foliar spray was low. In contrast, nHAP uptake was greater on adaxial leaf surfaces and favoured by higher surface tension. These findings demonstrate that aligning foliar nano particle formulations with their respective uptake pathways, spray properties, and leaf traits can substantially enhance nano‐particle uptake efficacy. This work provides new mechanistic insight to support more efficient and sustainable P management practices in potato production and broader agricultural systems.

## Summary statement

Nano‐hydroxyapatite particles enter potato leaves across stomata, while phosphate ions primarily enter across the cuticle. Their uptake efficiency can be equally high, but depends on the interaction between surface tension, leaf topography, leaf polarity, and environmental factors.

## 1 Introduction

Phosphorus (P) fertilisation in crop production is highly inefficient, contributing to various adverse environmental effects. Therefore, improvements in P uptake efficiency (PUE) within crop production constitutes one of the most acute challenges within the agricultural sector.

Potato plants (*Solanum tuberosum* L.) are of special interest when it comes to PUE improvements: Being the 6th most abundant crop in the world in 2023, potato plants have a shallow root system combined with a low root density (Fixen and Bruulsema 2014; FAO 2024). Therefore, potato plants are characterised by a poor ability to access P pools in the soil while displaying positive yield responses even at high P fertilisation rates (Fixen and Bruulsema 2014; Thornton 2020). On‐farm management strategies such as fertiliser placement (Koch et al. 2019; Cui et al. 2020), or biostimulant applications (Caradonia et al. 2021) have not yet resulted in environmentally and economically satisfactory results (Davenport et al. 2005; Elser and Bennett 2011; Withers et al. 2019). Thus, a foliar fertilisation strategy, where the soil is bypassed, seems like a meaningful alternative in order to increase the PUE.

However, foliar application of P salts, such as phosphoric acid, potassium‐ or ammonium phosphates, at agronomically relevant dosages is challenged by the high risk of leaf scorching (necrosis) (Peirce et al. 2019), which in turn limits tuber yields (Allison et al. 2002). A promising approach is the foliar application of a nano‐hydroxyapatite particle (nHAP) suspension: While exhibiting a weaker osmotic stress to the plant, the nHAP physical and chemical properties can be tailored to allow for foliar uptake (Husted et al. 2023). Cellular stimuli, including the low pH value of the cell wall, may allow for a slow and sustained release of Pi in accordance with the plant needs.

When a fertiliser droplet is deposited to the leaf surface, it must first adhere to the cuticle, then wet the area surrounding the droplet and spread. Stronger adherence and wetting occur upon stronger interaction between solution and surface. The surface free energy (γ) is a measure for the strength of this interaction. It depends on the physicochemical properties of the solution, as well as on the chemical and morphological leaf surface characteristics (Fernández and Khayet 2015; Fernández et al. 2021). Leaf surfaces tend to be more wettable with the presence of hydrophilic trichomes and when it contains patches of polar cuticle components such as hemicellulose and pectin (Fernández et al. 2014; Fernández and Khayet 2015; Fernández et al. 2016; Arsic et al. 2020; Guzmán‐Delgado et al. 2021). Leaf wetting can be promoted by adding surfactants and humectants to the spray solution. A lower surface tension of the droplet allows a faster spreading and improved contact between solvent and the waxy cuticle (Peirce et al. 2019). In general, a stronger interaction between solution and leaf surface are believed to be associated with higher foliar uptake of solutes and particles (Fernández and Khayet 2015).

As a next step, mineral ions, compounds, or dispersed particles need to cross the foliar barrier either by crossing the cuticle, or by entering through structures such as hydathodes, trichomes, or stomata. Leaf surface morphology, cuticle composition, and abundance of trichomes and stomata varies greatly between species, growth stages, leaf polarity (adaxial or abaxial), and nutritional status (Eichert and Goldbach 2008; Fernández et al. 2014; Fernández et al. 2017; Henningsen et al. 2023). The leaf cuticle is a waxy layer, which prevents water loss and solute leaching from the plant, and thus apparently prevents leaf penetration by water, solutes and pathogens (Fernández et al. 2017; Fernández et al. 2021). Despite this, penetration of solutes has been observed both through isolated and intact cuticles, with and without stomata in a variety of plants (Schonherr 2006; Berry et al. 2019; Guzmán‐Delgado et al. 2021). Small non‐polar molecules such as urea can diffuse through the cuticle, a route referred to as the hydrophobic pathway (Eichert and Goldbach 2008; Eichert et al. 2008). However, the cuticle is not only hydrophobic: Polar compounds such as hemicelluloses, pectin and lignin can stretch from the primary cell wall into the outer wax layers and allow water molecule sequestration within the cuticle under humid conditions. This process is believed to induce transient hydrophilic pore formation (Fernández et al. 2017; Arsic et al. 2020). It is a theoretical concept which is still being debated, but it would explain the cuticular uptake of ions observed in previous studies. For instance, P_i_ has been shown to cross the cuticle of barley in leaf regions with fibre cells, where pectin‐rich cell walls were believed to swell upon hydration, facilitating apoplastic transport (Arsic et al. 2020). A similar cuticular penetration of water in potato could not be observed (Frank et al. 2025). However, the cuticular pathway appears to be highly size restrictive and only available for small, non‐polar compounds, free ions and the smallest nanomaterials, as the estimated size exclusion limit seems to be below 5 nm (Eichert and Goldbach 2008; Fernández et al. 2017; Fernández et al. 2021).

Larger and especially polar particles are more likely to penetrate *via* the stomata, as stomatal presence, density and aperture have repeatedly been shown to promote uptake of foliar solutes and nanoparticles (Eichert et al. 2008; Kaiser 2014; Avellan et al. 2019; Husted et al. 2023). The stomatal architecture and hydrophobicity usually prevents penetration of solutions with a surface tension above a certain threshold estimated to 30 mN m^−1^ (Schönherr and Bukovac 1972; Stevens 2006). A lower surface tension can be achieved by adding specific surfactants to the applied solution, which allows to overcome the stomatal barrier within minutes to hours (Stevens 2006; Burkhardt et al. 2012; Hu et al. 2020). It remains unclear whether stomatal closure is capable of fully preventing this process. At higher surface tensions, diffusion along a thin water continuum spanning from the atmosphere along the guard cell surface into the substomatal cavity may take place. The formation of such a liquid continuum has been coined as “hydraulic activation of stomata” (HAS) and has been found to involve only about 10% of all stomata (Eichert and Burkhardt 2001; Eichert et al. 2008; Burkhardt et al. 2012). HAS can be triggered by deposition of hygroscopic aerosols such as salts, dust, nanoparticles (NPs) or adjuvants, and is reported to require weeks to establish (Burkhardt 2010; Burkhardt et al. 2012; Burkhardt et al. 2018; Grantz et al. 2018; Vega et al. 2023). Surprisingly, HAS in potato leaves establishes within 3 h at high humidity in the majority of open stomata covered by a water droplet at ɣ = 70 mN m^‐1^ (Frank et al. 2025). The efficiency of HAS as a transport pathway for fertiliser NPs is still unknown. Furthermore it is unclear, whether HAS is strictly necessary in order to facilitate stomatal uptake.

Generally, stomatal uptake is believed to be higher on the abaxial (lower) side of the leaf than on the adaxial (upper) side. It increases with stomatal density (Eichert and Burkhardt 2001) and opening (Eichert and Goldbach 2008; Eichert et al. 2008; Burkhardt et al. 2012; Kaiser 2014), relative air humidity (RH) and rewetting (Eichert et al. 2008; Fernández et al. 2020; Schreel and Steppe 2020; Fernández et al. 2021; Barlas et al. 2023), the concentration gradient between droplet and foliar tissue (Burkhardt et al. 2012), and time. Solution properties associated with higher uptake are low surface tension, positive charge, and hygroscopicity (Burkhardt 2010; Avellan et al. 2019; Hu et al. 2020; Eichert and Fernández 2023), although specific leaf topographies, especially in potato, might result in more efficient uptake at higher surface tensions (Frank et al. 2025).

To date, we know little about how formulations of foliar sprays can be optimised with respect to leaf chemical and morphological properties to ensure best possible surface wetting and retention. Simultaneously, improved surface wetting might result in faster spreading and thus drying of the solution on the surface – a delicate balance requiring more research. While hydrophilic cuticular uptake of P_i_ has been observed along leaf veins in barley (monocot) (Arsic et al. 2020), this has so far not been shown for a dicot. It remains largely unknown how surfactant choice and leaf surface properties affect the contribution and extent of cuticular and stomatal pathway, and whether trichomes contribute to NP uptake. It is unclear whether diffusion or mass flow are contributing most to foliar uptake of ions and particles through stomata, and under which conditions one or the other is favoured. This might have strong implication for the timing of fertiliser application. Finally, the uptake efficiency of P_i_ and P containing nanoparticles (nHAP) has never been investigated in field grown plants. Therefore, it is unclear whether foliar fertilisation with nHAPs can be sufficiently efficient compared with Pi or how it can be optimised in a plant‐specific way. In the future, it will be essential to fill these research gaps to make P fertilisation practices more efficient and sustainable.

We test the following hypotheses:

1. Pi is capable of penetrating potato leaves across the leaf cuticle, while nHAPs predominantly enter the leaves across stomata.
2. The density, physical and chemical properties of potential uptake routes (cuticle, stomata, trichomes) vary with leaf age, polarity, and P nutritional status.
3. Once the nHAP formulation has been optimised according to the leaf surface properties and the dominating uptake pathway(‐s), foliar PUE values may significantly exceed those typically found in soil based P fertilisation.

In order to address these hypotheses, we investigate changes in cuticle composition, leaf surface topography and trichome and stomatal density under varying P nutritional status, leaf age and leaf polarity in potato plants. We follow a mechanistic approach to disentangle the influence of chemical and physical leaf surface properties, surfactant use, and droplet surface tension on the wetting of potato leaves. Various bioimaging techniques were employed to observe whether P_i_ and nHAP enter into the leaf tissue *via* the cuticular pathways, stomata, or trichomes, and how these relate to the properties of leaf and solution. Finally, a field trial using ^33^P tracers was conducted, to compare foliar P uptake under laboratory and green house conditions with a real life scenario in a commercial potato field.

## 2 Materials and Methods

### 2.1 Plant Growth in the Greenhouse

Potatoes seeds (*Solanum tuberosum* L. c.v Assol, early cultivar) were obtained from the market (SeedEra, Ukraine) and germinated in vermiculite watered with double deionized water for 18 days (d) until root development. At leaf stage3‐4 the seedlings were transplanted to hydroponics. Plants for the experiments were propagated from the hydroponic culture by taking cuttings (tops of side shoots with 4 leaves), which were allowed to root in vermiculite for 10 d, then transplanted to a new set of hydroponics for 3–4 weeks before start of the experiments. The greenhouse was generally operated at 350‐400 µmol m^−2^ s^−1^ PAR, using a 16: 8 h (h) day: night cycle. During seed germination and propagation temperature was 25°C (85% RH) and during hydroponic growth reduced to 20: 15°C day: night (65% RH). Plants cultivated for the ^33^P tracer experiment were grown in a climate chamber at 350 µmol m^−2^ s^−1^, 20: 15°C and 55% RH.

Three plants were grown in each 5 L aerated hydroponic container, exposed to either P deficiency (P−) or P sufficiency (P + ). Three independent replicates of each treatment were used. Nutrient concentrations in the solutions were P: 0.1 mM (P−) or 0.8 mM (P + ), N: 6.0 mM, K: 2 mM, S: 1.6 mM, Mg: 1.2 mM, Na: 0.2 mM, Cl: 0.5 mM, Ca: 1.8 mM, Fe: 0.1 mM, Mn: 2 µM, Zn: 1.4 µM, Cu: 1.6 µM, B: 4 µM, Mo: 1.6 µM. Solutions were replenished every 7 d and pH was maintained at 5.5–6.5 by regular adjustments.

P deficient potato plants displayed typical P deficiency symptoms, including darker and smaller leaves, anthocyanosis, and stunted growth (Supporting Information: Figure S1). Chlorophyll *a* fluorescence transients (OJIP analysis) confirmed that plants were P deficient compared to plants cultivated with ample P (Supporting Information: Figure S2).

### 2.2 Scanning Electron Microscopy (SEM)

Leaf pieces of approx. 5 mm × 5 mm were fixed for 4 h in Karnovsky's fixative (5% v/v glutaraldehyde, 4% v/v paraformaldehyde, 0.1 M sodium cacodylate buffer, pH7.3), washed in buffer and water, dehydrated in ascending concentrations of acetone, and transferred from 100% water‐free acetone prior to critical point drying in an EMS 850 CP drier (Hatfield, Pennsylvania, US). Specimens were mounted onto metal stubs, sputter‐coated with 6 nm gold in an EM ACE200 (Leica Microsystems, Wetzlar, Germany) automated sputter coater and viewed either in a Quanta 200 SEM (FEI CompanyTM, Hillsboro, Oregon, US) or in a Quanta 3D FEG SEM (FEI CompanyTM). Stomata and trichomes were counted on 4‐7 repeated micrographs obtained from different areas of the same leaf. Images shown in this study were performed by Dr. Victoria Fernandez at the Spanish National Centre for Electron Microscopy (ICTS) in Madrid, Spain.

### 2.3 Transmission Electron Microscopy (TEM)

Leaves for Transmission Electron Microscopy (TEM) were fixed in glutaraldehyde (2.5%) and paraformaldehyde (4%) for 3 h, then rinsed in cold phosphate buffer (pH 7.2). Tissues were post‐fixed in a 1:1 aqueous solution of 2% osmium tetroxide (TAAB Laboratories, Berks, UK) and 3% potassium ferrocyanide (Sigma‐Aldrich, Burlington, Massachusetts, US), rinsed in water and dehydrated in ascending concentrations of acetone. Samples were gradually embedded in acetone‐Spurr's resin (TAAB Laboratories Equipment Ltd., Berkshire, UK) mixtures (3:1 for 2 h, 1:1 for 2 h, 1:3 for 3 h (v:v)) and finally pure resin. Ultra‐thin sections (obtained with a Leica Ultracut E, Leica Microsystems, Wetzlar, GER) were consequently cut, mounted on nickel grids and post‐stained with Reynolds lead citrate (EMS) for 5 min prior to sample analysis. Petal sections were observed with a Jeol 1010 TEM (Jeol Ltd., Tokyo, JP, at 80 kV) equipped with a CCD Megaview camera.

### 2.4 Coriphosphine‐O Staining and Fluorescence Microscopy

Youngest fully evolved leaf (YFEL) cuts (approx. 4 mm x 10 mm) of P sufficient and P deficient 4 week old potato plants were used. All further steps including imaging occurred as described in (Arsic et al. 2020). Depth focus in uneven areas was obtained by producing a series of microscopic pictures with varying depth focus followed by processing the images using the software Helicon Focus 8 (Helicon Soft Ltd., Kharkiv, Ukraine). Contrast and brightness were slightly adjusted using FIJI (Schindelin et al. 2012).

### 2.5 Attenuated Total Reflectance – Fourrier Transformed Infra‐red (ATR‐FTIR) Spectroscopy

The surface biopolymer composition was analysed using ATR‐FTIR spectroscopy, with a Nicolet 6700 FT‐IR equipped with a Pike Technologies GladiATR diamond spectrometer (Thermo Scientific, Waltham, Massachusetts, US). Fresh leaves of leaf positions 2, 4 and 8 from P+ and P− plants after 21 d in hydroponics were each measured 5 times on the abaxial and adaxial leaf sides. Data was analysed in MATLAB, where a PCA was performed. Wax:carbohydrate ratios were calculated as the sum of the areas under peaks 2915 and 2850 cm^‐1^ divided by the area under the peak at 1033 cm^‐1^.

### 2.6 Contact Angles and Surface Tensions

Contact angles and surface tensions was measured with an optical tensiometer (Thetaflow, Biolin Scientific, Gothenburg, SE). All measurements were performed on freshly prepared solutions. Surface tension was measured by pendant drop method, on four 6–10 µL droplets, and the stable values are reported. Contact angles were measured on leaf sections mounted on glass slides with double sided tape. 3‐5 µl droplets were applied to the surface, observed over 180 s (s), and the contact angle calculated by the software using the Young‐Laplace model. To compare droplet spreading on plants of differing P status, values were recorded for 15 s after droplet deposition and averages were compared statistically (see below).

### 2.7 Doped Nano Hydroxyapatite (NHAP) Synthesis

The Ce/Sr and ^33^P labelled nHAPs were synthesised as follows: CaCl_2_ * 6 H_2_O (final concentration = 270 mM, VWR, Radnor, Pennsylvania, US) and NH_4_‐citrate tribasic (final *c* = 100 mM, Sigma Aldrich, Massachusetts, US) were mixed on a magnet stirrer at 600 rpm. For Ce doped nHAPs, Ca was replaced by Ce at a 1:100 molar ratio with Ce(NO_3_)_3_ * 6 H_2_O (Sigma Aldrich, Massachusetts, US). K_2_HPO_4_ (final *c* = 161 mM, VWR, Pennsylvania, US) was added to the mixture under continuous stirring and kept on the magnet stirrer for 24 h while the particles matured. The pH of the components was adjusted to pH 9 with NH_4_OH (Sigma‐Aldrich, Massachusetts, US) before mixing. For ^33^P spiked nHAPs, KH_2_PO_4_ was partly replaced by radioactive phosphoric acid (Hartmann Analytics, Braunschweig, GER) to achieve 1.369 MBq ml^‐1^. Excess ions and NH_4_^+^ was removed by dialysis after particle maturation: 50 ml nHAP suspension was floated in a 3.5 kDa snakeskin dialysis tube (Sigma Aldrich, Massachusetts, US) in a 5 L 100 mM NH_4_‐citrate bath for 24 h under gentle stirring. Size and charge were measured with Dynamic light scattering (DLS, Malvern Zetasizer, Worcestershire, UK).

### 2.8 Nanoparticle Characterisation

The particles used in this study have been characterised extensively in (Minutello et al. 2026). In brief: TEM micrographs showed needle shaped particles with median dimensions of 33.6 nm x 5.3 nm, and an aspect ratio of 6.3. DLS measurements revealed a good stability in suspension, and a hydrodynamic diameter of 32.9 nm. The polydispersity index indicated a narrow size distribution of 0.092, similar to the TEM results. The zeta potential was ζ = −28 mV, indicating repulsion between particles. Small angle X‐ray scattering (SAXS) data had a best fit with an elliptical cylinder model, yielding about 70 nm in length and 1.5‐10 nm in radius. Ionic coupled plasma mass spectrometry (ICP‐MS) after particle dialysis confirmed a P concentration of about 4.6 g L^‐1^, a Ca:P molar ratio of 1.68–1.71, corresponding to hydroxyapatite, and the successful incorporation of the elemental tracers. Find all details in (Minutello et al. 2026).

### 2.9 X‐Ray Micro Computed Tomography (X‐Ray ΜCT)

All experimental procedures and data processing are described extensively in (Frank et al. 2025). The data presented here originates from the same experiment as in the reference. In brief: 14 days old potato plants were incubated in < 50 ppm CO_2_ and > 90% RH until stomata were open. Droplets containing 150 mM of the contrast agent iohexole were added at different surface tensions to adaxial and abaxial sides of horizontally aligned YFELs. X‐ray µCT scans were conducted at different time points after droplet application at the DanMAX beamline of the MAXIV synchrotron in Lund, Sweden. Control: No droplet. Adaxial scan: ɣ = 70 mN m^‐1^, imaged after 5 h. Abaxial scan: ɣ = 40 mN m^‐1^, imaged after 3.5 h. Details are describedin Frank et al. 2025.

### 2.10 Confocal Laser Scanning Microscopy (CLSM)

The amphiphilic fluorescent dye DiI was diluted in DMSO (Sigma Aldrich, Massachusetts, US) in a concentration of 300 mg L^‐1^. 50 µl of the solution was diluted 20x in milliQ water adjusted in surface tension to ɣ = 50 mN m^‐1^ by using the surfactant Silwet Gold (HD 2412, DK) at a final concentration of 0.005% w/v. Control: no dye, only milliQ and surfactant. A 10 µl droplet was deposited on a potato YFEL and incubated in < 50 ppm CO_2_ and > 90% RH as described in (Frank et al. 2025). 2 h after droplet application, the treated leaf area was imaged in water using a Leica Stellaris 8 CLSM (Leica Biosystems, Deer Park, US), and a 20x glycerine immersion objective lens. System settings: Excitation laser at 550 nm with 2% intensity. DiI fluorescence signal detected at 560–610 nm with a HyD X2 detector and a gain of 30. Chlorophyll autofluorescence detected at 700 – 860 nm, HyD S 3, gain 90. Pinhole: 1 AU. All images were processed in the same way in LAS X Office version 1.4.4.26810. CLSM images represent total vertical projections of z‐stacks (z‐step size 3 µm), with subsequently assigned colours (DiI: cyan scale 5‐40, autofluorescence: red scale 120‐255).

### 2.11 Laser Ablation ‐ Inductively Coupled Plasma ‐ Mass Spectrometry (LA‐ICP‐MS)

For LA‐ICP‐MS, YFELs of potato plants rooted for 2 weeks in vermiculite with nutrient solution were used. 30 min prior to the treatment, plants were exposed to 250 PAR, < 50 ppm CO_2_ and > 90% RH as described in (Frank et al. 2025) in order to force stomata open. Droplets with 10 µl solution were deposited on each side of the midrib of the outer leaflet of the YFEL. Treatments: nHAP_Ce_ (γ = 68 ± 2 mN m^‐1^, n = 2), nHAP_Ce_ + 0.01% Silwet Gold (γ = 26 ± 1 mN m^‐1^, n = 2). Control: nHA*p* + 0.01% Silwet Gold (γ = 26 ± 1 mN m^‐1^, n = 2), no spiking. Samples of the treated leaflet and the according petiole were taken after 5 h as described below. To investigate P_i_ uptake, 0.2 mM KH_2_PO_4_ (VWR, Pennsylvania, US) spiked with 1 mM Na_3_VO_4_ (Sigma Aldrich, Massachusetts, US) was applied at pH 6 with and without 0.01% Silwet Gold. Control: 0.2 mM KH_2_PO_4_ (VWR, Pennsylvania, US) with 0.01% Silwet Gold, pH 6. 5 h after droplet application, 5 mm x 1 cm leaf sections or 1 cm long petiole pieces were cut out using a scalpel and embedded in OCT by freezing down to −60°C within 30 s using cold hexane without allowing direct contact between sample and hexane. Samples were stored at ‐20°C until cutting in a cryotome (Cryostat CM3050 S, Leica Biosystems, Deer Park, US) at ‐25°C. 14 µm slices were transferred to Cryofilm type IIC(9) (Section‐lab Co. Ltd., Yokohama, Japan) and fixed on object slides with double sided tape. Leaf sections were dried by storing them over night in the cryotome chamber under ventilation and kept in a desiccator until processing in LA‐ICP‐MS. Light microscopy images were taken with a Leica microscope (ocular 10x/22, objective lens 10x/0.25, Leica Biosystems, Deer Park, US), before elemental maps of ^24^Mg, ^140^Ce, and ^51^V were produced. Samples were ablated with a 193 mm ArF excimer laser (Iridia, Teledyne photon systems, Thousand Oaks, US) equipped with a Cobalt cell (Teledyne photon systems). Laser settings for nHAP_Ce_ samples: spot size 3 µm circle, fluence 1,7 J cm^‐2^, fixed dosage 5, repetition rate 300 Hz. For vanadate samples: fluence 1,5 J cm^−2^, fixed dosage 6, repetition rate 200 Hz. Drifts in ICP‐MS sensitivity were monitored using a NIST612 glass standard (NIST; National Institute for Standards and Technology, SC, USA). Helium gas was used as a transport gas to the ICP‐QQQ‐MS 8900 (Agilent, Santa Clara, US). ICP‐MS settings: no‐gas mode, integration times: 3 ms for ^24^Mg, 12 ms for ^140^Ce with a total scan cycle of 25 ms. Integration times for vanadate treatments: 4 ms for ^24^Mg, 15 ms ^51^V. Image reconstructions and overlays were produced in HDIP (Teledyne CETAC Technologies) and figures were set together in InkScape.

### 2.12 ^33^P Radioisotope Treatments in the Climate Chamber

Foliar solutions of potassium phosphate (130 mM P, as 15% KH_2_PO_4_ and nHAP (15 g/l corresponding to 0.13 M P, pH 8) were spiked with ^33^P ([^33^P] phosphoric acid, Hartmann analytic, Braunschweig, GER) to an activity concentration of 1.37 MBq ml^‐1^. For climate chamber experiment: 0.02% and 0.05% Silwet Gold (γ = 33 mN m^‐1^ and γ = 22 mN m^‐1^) was added to the solutions. Surface tensions were determined on non‐radioactive solutions using an optical tensiometer (Theta Flow, Biolin Scientific, Gothenburg, SE). Foliar solutions were applied at early flowering stage, which was after 28 d in hydroponics in growth chambers. Solutions were applied to the 4th leaf (YFEL). 74 µl was deposited on the outermost leaflet. After deposition, the solutions were spread with a Lazy‐L cell culture spreader to cover the complete adaxial or abaxial leaf surface. 4 replicate plants were harvested 7 d after treatment. The treated leaves were washed 5 times in 0.1% Tween 20 before drying. The treated fraction consists of the YFEL. The shoot above the YFEL was designated as the “younger” fraction, while the “older” tissue was all below the YFEL and the “roots” refer to the roots.

### 2.13 ^33^P Radioisotope Treatments in the Field

In the field, the same solutions were applied as in the climate chamber experiment. Surface tensions were adjusted to γ = 50, 40, 30, and 20 mN m^−1^ by mixing with 0.006%, 0.008%, 0.02% and 0.05% Silwet Gold, respectively. Control: 50 mM NH_4_‐citrate at γ = 20 mN m^−1^. 5 droplets of 10 µl solution were applied to the adaxial side of the 5 outermost leaflets of the YFEL. 4 replicate plants were harvested 7 d after treatment. The treated leaves were washed 5 times in 2 l of 0.1% Tween 20 before drying. The treated fraction consists of the YFEL. The plants were separated into the tip above the treated leaf, the treated YFEL, the treated tiller, roots, tubers, and other tillers grown from the same mother potato.

The field is located in 55°29′53.3″N 9°12′02.9″E, 6600 Vejen, Jutland, DK (Mark 520) and cultivated with the potato variety Kuras. The trial was conducted in the late growth stage within the first half of August 2024 (BBCH growth stage 635: full flowering). Treated replicate field rows were interspaced with two non‐treated rows (approx. 2 m distance), and all treated plants were interspaced with two non‐treated plants (approx. 1.5 m distance). Treatments were randomised along rows. Hourly resolved precipitation data for Vejen was obtained from the Danish Meteorological Institute (DMI). Leaf wetness and temperature was monitored by installing a PHYTOS 31 leaf wetness sensor on a ZL6 data logger (METER, Pullman, US) at the height of YFELs within the canopy. Data was logged in an interval of 15 min.

### 2.14 Radioactivity Measurements

Plant samples were dried at 60°C for a minimum of 72 h until completely dry, then pulverised using zirconium balls. Dry plant samples were digested in an Ultrawave (Milestone Srl, Sorisole, IT), with 100 mg dried plant material, 2.5 ml 70% HNO_3_ and 1 ml 37% H_2_O_2_, heated to 240°C under 8 MPa for 15 min, and diluted to 50 ml in miliQ water. For the field trial: Tubers were freeze‐dried before milling and for all samples, 200 mg of dried plant material was digested and diluted to 30 ml.

To measure ^33^P activity in treated samples and untreated controls, 5 ml diluted plant sample digest were mixed with 15 ml scintillation fluid Ultima Gold XR (Sigma‐Aldrich, Massachusetts, US) and measured with liquid scintillation counting on a Tri‐Carb® 2910TR; PerkinElmer, Shelton, Connecticut, US. Fractions of total applied amounts were calculated from calibration curves made from dilutions of the applied solutions. The LOD was calculated based on blank samples as $\bar{x}$ + 3δ and substracted from all reported values. Based on 4 biological replicate samples, 90% confidence intervals were constructed for each treatment, and outliers were removed accordingly. Bar plots show averages and standard errors of outlier‐free data sets (see below).

### 2.15 Phosphor Screen Imaging

YFELs of potato plants treated in the field were harvested after 7 d, washed in 4 subsequent buckets with milliQ water, and were laid flat onto a working bench after air drying. Molecular Dynamics Storage Phosphor Screen (Amersham Biosciences, Buckinghamshire, UK) were wrapped in household plastic foil for protection and were exposed to the sample for 48 h. Phosphor screens were scanned in an Amersham Typhoon IP (Amersham Bioscinces) scanner at a pixel size of 50 µm^2^. In ImageJ (Schindelin et al. 2012), greyscale images were adjusted in contrast and were given a colour scale.

### 2.16 Statistics

Bar plots show means of single measurements, and error bars display standard errors. Letters above error bars derive from a pairwise comparison of several data sets using the Šidák test method in R. For the data analysis, single data points were removed as outliers if they were either:

≤Q25−1.5∗(Q75−Q25)or ≥Q75+1.5∗(Q75−Q25), with Q25 and Q75 being the 25% and 75% quantiles, respectively.

## 3 Results

### 3.1 Leaf Surface and Tissue Characteristics

**Figure 1:**
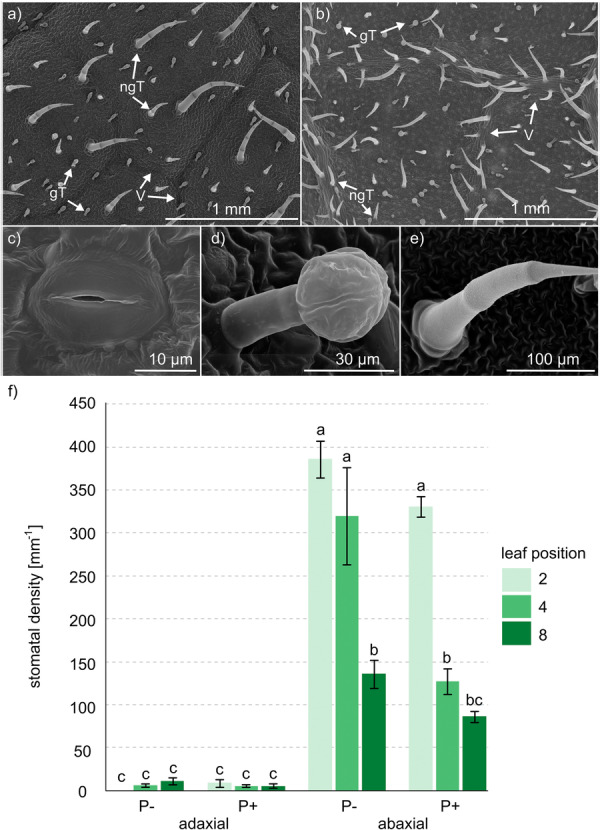
Leaf surface structures on youngest fully evolved leaves (YFELs) of 3‐week‐old potato plants. (a) to (e) SEM micrographs of P sufficient YFEL surfaces. (a) Adaxial, (b) abaxial leaf side. (c) Stoma, (d) glandular trichome, (e) non‐glandular trichome. Labels: V: vein, gT: glandular trichome, ngT: non‐glandular trichome. (f) Density of stomata on adaxial and abaxial leaf sides of leaf positions 2 (youngest leaf), 4 (YFEL), and 8 (oldest leaf) of P deficient (P−) and P sufficient (P + ) potato plants. The structures were counted in 4–7 randomly chosen areas on the leaf on SEM micrographs. Error bars indicate standard deviations, and letters indicate groups of significance based on an α ≤ 0.05 significance level (Šidák test).

Scanning Electron Microscopy (SEM) imaging showed that stomatal guard cells were topographically elevated above the surrounding leaf surface (Figure 1c). Veins appeared as depressions from the adaxial leaf side, while appearing as elevations on the abaxial side (Figure 1a,b).

Stomata were uniformly distributed between the abaxial veins (Figure 1b), while being concentrated along the veins on the adaxial leaf side (Figure 1a). YFELs had 330 ± 29 stomata mm^‐2^ on the abaxial leaf side, but only 8 ± 8 stomata mm^‐2^ on the adaxial leaf side (Figure 1f). While the leaf position did not influence the stomatal density on the adaxial leaf side, it drastically decreased with leaf age on the abaxial leaf side (Figure 1f).

P deficiency led to an increased stomatal density on the abaxial side of the youngest leaves, but did not result in changes on the adaxial leaf side. Strikingly, the YFEL (leaf position 4) had 319 ± 113 stomata mm^‐2^ in P‐ treatments, but only 127 ± 34 stomata mm^‐2^ in P+ treatments, corresponding to an almost threefold higher stomatal density under P deficiency (Figure 1f).

Referring to the trichome types described by (Papierowska et al. 2020), non‐glandular trichomes (ngT) type II (Figure 1e), and glandular trichomes (gT) type VII (Figure 1d) and type VI were observed on both sides of the leaf. On the abaxial leaf side, ngT were most numerous and concentrated along the veins, while gT were evenly distributed between veins (Figure 1b). ngT were generally less numerous, but larger on the adaxial side (Figure 1a and Supporting Information: Figure S3). In P+ plants, no differences in the trichome densities were found between the two leaf sides, and leaf positions did not significantly alter the trichome density (Supporting Information: Figure S3). P deficiency drastically increased the abundance of trichomes on young leaves compared to P+ plants, and trichome densities decreased with leaf age (Supporting Information: Figure S3). ngT were more abundant on the abaxial side of P‐ leaves (Supporting Information: Figure S3b).

Attenuated Total Reflectance – Fourier Transformed Infra‐red Spectroscopy (ATR‐FTIR) was used in order to investigate differences in the chemical composition on both leaf surfaces of potato leaves (P− and P + ) at different leaf positions. Younger leaves, the adaxial side, and P deficiency coincided with higher carbohydrate and wax contents compared to older leaves, the abaxial leaf side, and P sufficiency. However, the hydrophobic wax to hydrophilic carbohydrate ratios were constant among all treatments, and thus not explanatory for leaf‐solution interactions (Supporting Information: Figure S4).

**Figure 2:**
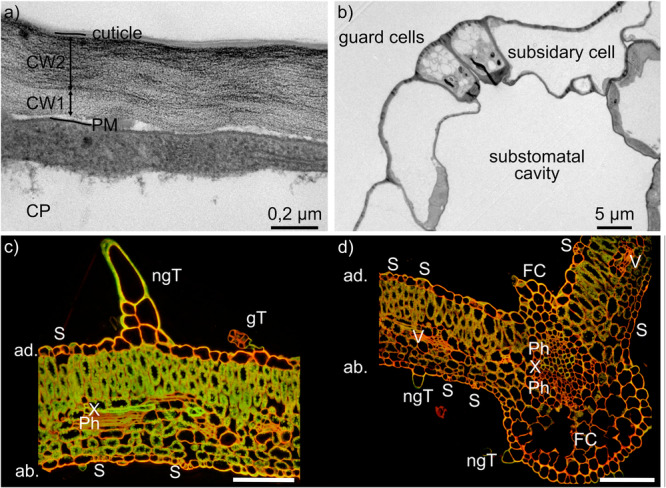
Morphology of P deficient potato leaves. (a) and (b): TEM micrographs. (c) and (d): Coriphosphine O stained YFEL cross sections imaged by fluorescence microscopy. ad: adaxial leaf side, CW1: primary cell wall, CW2: secondary cell wall, PM: plasma membrane, CP: cytoplasm, gT: glandular trichome type VI, ngT non‐glandular trichome, S: stoma, Ph: phloem, V: vein, X: xylem, FC: fibre cells. Scale bars in (c) and (d) correspond to 100 µm. [Color figure can be viewed at wileyonlinelibrary.com]

Transmission electron microscopy (TEM) revealed that the cuticle in potato leaves varied between 30 nm and 55 nm in thickness, with no evident impact of the P status or differences between adaxial and abaxial cuticles. The cell walls of epidermal cells were 500–600 nm thick (Figure 2a). Stomatal openings protruded from the surrounding leaf surface topographically in TEM micrographs and fluorescence microscopy (FM) images, confirming the findings from SEM in Figure 1. Optically, closed stomata appeared to be tightly sealed. No open stomata were observed in the acquired images (Figure 2b). No differences were observed in cuticle, cell wall or stomatal morphology between plants of differing P status. FM of fixed, Coriphosphine O stained, YFEL cross sections of P− plants revealed the multicellular organisation of trichomes (Figure 2c,d). While ngT had a pectin rich multicellular base, gT type VI had a pectin rich multicellular head. In addition, all cuticular cells appeared to have pectin‐rich cell walls, including stomatal guard cells. Sub‐stomatal cavities were clearly visible as hollow spaces. However, occasional diagonal or longitudinal cutting of stomata in their periphery made it difficult in some cases to localise a stoma.

### 3.2 Leaf Wetting

Droplets deposited on adaxial leaf surfaces spread out faster than droplets deposited on the abaxial leaf surface. However, the difference in leaf polarity became smaller as γ decreased (Supporting Information: Figure S5). Lower γ generally resulted in faster spreading of droplets on a wider leaf area (Supporting Information: Figure S5), but as a consequence droplets also dried out faster (data not shown).

### 3.3 Foliar Uptake Pathways of Nano‐Hydroxyapatite Particles

**Figure 3:**
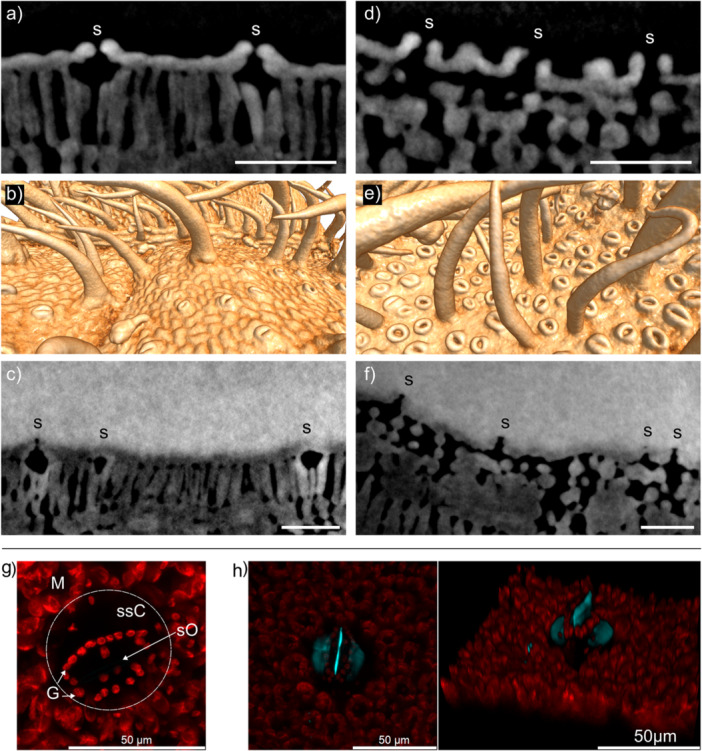
Adaxial versus abaxial penetration of open stomata by foliar liquids. (a)–(f) X‐ray µCT data. (g), (h) CLSM images (a)–(c) adaxial leaf side, (d), (e) and (g), (h) abaxial leaf side. a) virtual cross‐sections show that adaxial stomata were open under control conditions, with a shape we describe as ‘volcano shaped’, while (d) stomata on the abaxial side were open with a shape we describe as ‘tube shaped’. In (b) and (e), false colour stained 3D models illustrate the topographic differences that foliar liquids encounter on the (b) adaxial and on the (e) abaxial leaf side. (c) After 5 h, water (white) from a ɣ = 70 mN m^−1^ droplet penetrates open stomata (grey) on the adaxial leaf side. (f) In contrast, stomata on the abaxial leaf side are not penetrated by the liquid after 3.5 h at ɣ = 40 mN m^−1^. Instead, the stomatal pore space is filled with gas (black). (e) CLSM imaging occurred 2 h after droplet application at ɣ = 50 mN m^−1^. Red: Chlorophyll autofluorescence, corresponding to the localisation of chloroplasts. Cyan: DiI, an amphiphilic fluorescent dye. a) control, no dye. The image shows a total projection of z‐stacks. The lack of signal from the DiI channel indicates limited background originating from the leaf tissue or the surfactant. b) 5 µg L^−1^ DiI. The images are 3D reconstructions illustrating that the dye enters the sub‐stomatal cavity by moving along the guard cell surface. S: stoma, M: mesophyll, ssC: sub‐stomatal cavity, indicated by the circle. sO: stomatal opening. G: guard cells. Scale bars correspond to 50 µm. [Color figure can be viewed at wileyonlinelibrary.com]

Based on previous studies in scientific literature, it is likely that most NPs > 5 nm in size would penetrate the leaf by entering stomata along a water film. By using X‐ray µCT, we characterised the shape of open adaxial stomata as ‘volcano shaped’ and abaxial stomata as ‘tube shaped’ (Figure 3a,b,d,e). Recently, it was shown that HAS can occur in potato leaves adaxially at ɣ = 70 mN m^−1^ within 5 h after droplet deposition and is less likely to develop at low ɣ ((Frank et al. 2025), reproduced for ɣ = 70 mN m^−1^ in Figure 3c). We verified this finding with CLSM by choosing an intermediate surface tension of ɣ = 50 mN m^−1^ (Figure 3g,h). The control treatment revealed no surfactant related background signal (Figure 3g). Open stomata started being penetrated by the amphiphilic fluorescent dye DiI within 2 h after droplet deposition (Figure 3b and Supporting Information: Video S1). The dye was traced from the leaf surface along guard cell surface into the stomatal opening. It followed along the cell walls of subsidiary cells into the sub‐stomatal cavity, reaching the level of mesophyll cells as indicated by the signal originating from chlorophyll autofluorescence. This indicated that HAS had taken place in a fraction of the stomata. No leaf surface penetration by the dye was found in connection to other structures such as trichomes or veins. In contrast, ‘tube shaped’ stomata on the abaxial leaf side were not visibly penetrated by the overlying liquid at ɣ = 40 mN m^‐1^ after 3.5 h incubation. Instead, gas from within the leaf tissue appeared to push into the water film (Figure 3f).

nHAPs supposedly require stomata for foliar penetration, whereas P_i_ might as well enter *via* cuticle and/or trichomes. Therefore, we hypothesised that altering ɣ would affect uptake along these pathways in a contrasting manner. To address this, we conducted LA‐ICP‐MS on Ce spiked nHAPs (nHAP_Ce_) to compare their foliar uptake behaviour to V_i_ as a proxy for P_i_ at ɣ = 68 mN m^‐1^ *versus* ɣ = 22 mN m^‐1^.

**Figure 4:**
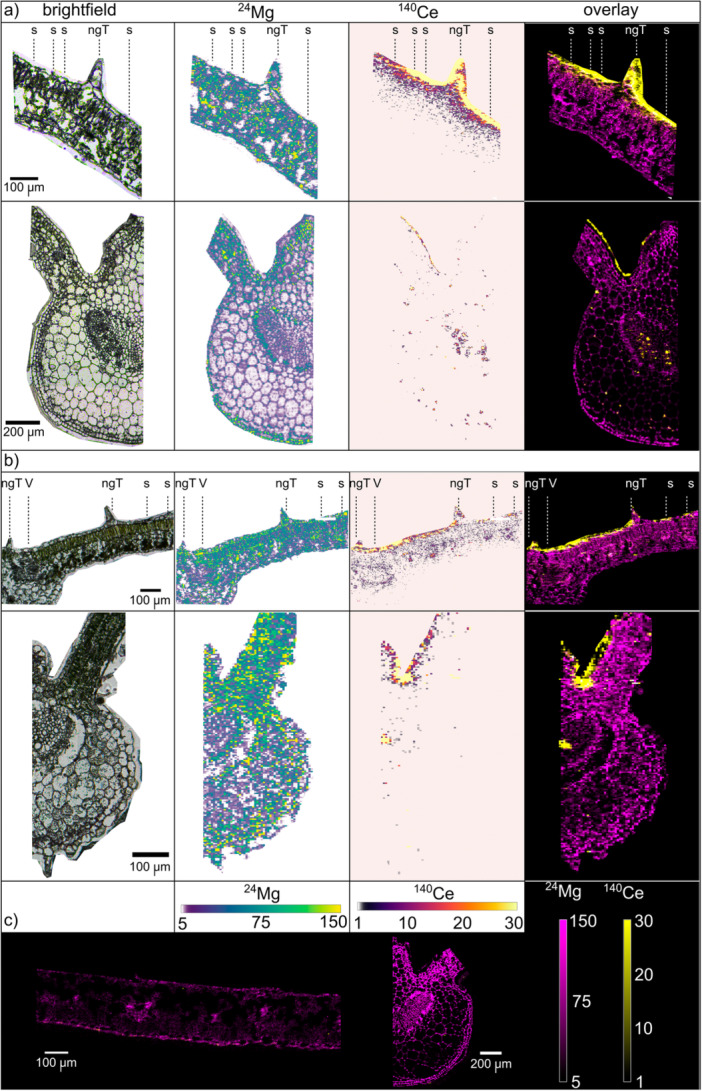
Light microscopy and LA‐ICP‐MS based elemental maps of nHAP_Ce_ treated YFELs and petiole cross‐sections. (a) nHAP_Ce_ at γ = 26 ± 1 mN m^‐1^. (b) nHAP_Ce_ at γ = 68 ± 2 mN m^‐1^, no surfactant. (c) control, nHAP at γ = 26 ± 1. Treated leaflets and petioles were harvested after 5 h at 250 PAR, < 50 ppm CO_2_ and > 90% RH. For each treatment, a cross‐section of the treated leaflet and the according petiole are shown. Irrespective of the droplet surface tension, nHAP_Ce_ entered the treated leaflet and were found in the connected petiole. S: stoma, ngT: non‐glandular trichome, v: vasculature. Note that the units of the colour scales correspond to ion counts by the ICP‐MS, but that the images are non‐quantitative by nature of the analysis technique. [Color figure can be viewed at wileyonlinelibrary.com]

Strikingly, irrespective of the surface tension, both in absence (Figure 4a) and in presence (Figure 4b) of the surfactant, the ^140^Ce signal was detected within the leaf tissue and the connected petiole 5 h after foliar droplet deposition. The term ‘within the leaf tissue’ is used here whenever the tracer signal overlayed with the ^24^Mg background map of the tissue section. Penetration by particles and/or ions occurred across the cuticle, stomata, and along the thick cell walls at the bases of non‐glandular trichomes (Figure 4a,b). Cuticular uptake was not specific to the location of veins, where fibre cells originating from the bundle sheath extension are located (Figure 4b).

5 h after droplet deposition, ^140^Ce was found inside the vasculature of the connected petiole. Phloem cells were small and interspaced with parenchymal cells and xylem vessels (Figure 4a,b, light microscopy). As the resolution of the data did not allow to clearly distinguish between phloem cells and xylem vessels, potential transport mechanisms remain unclear.

To study foliar Pi uptake, we used vanadate (Vi) as a tracer which has a zero background in leaf tissue and was found to be a suitable proxy for Pi in previous studies (Arsic et al. 2020). V_i_ showed marked differences in foliar uptake pathways after 24 h compared with nHAP_Ce_ (Supporting Information: Figure S6). Only when vanadate was applied, significant signal was collected (Supporting Information: Figure S6a–e).

At γ = 68 mN m^‐1^, the ^51^V signal collected appeared to be weaker than at γ = 24 mN m^‐1^ (compare Figure f6a‐b to Supporting Information: Figure S6c‐e). In both cases, ^51^V was taken up into the leaf tissue all across the leaf surface including the cuticle and bases of ngT, but concentrated specifically in gT type VII heads and around stomatal guard cells (Supporting Information: Figure S6a and e). ^51^V uptake appeared to occur independently of stomata (Supporting Information: Figure S6c,d). It was not possible to identify a specific uptake pathway. Unlike nHAP_Ce_ (Figure 4a,b), ^51^V did not accumulate within the vasculature (Supporting Information: Figure S6b and d) after foliar penetration, but distributed all across the leaf cross‐section.

### 3.4 Quantitative Uptake Efficiency of Foliar P_I_ Versus NHAP at Varying Surface Tensions

**Figure 5:**
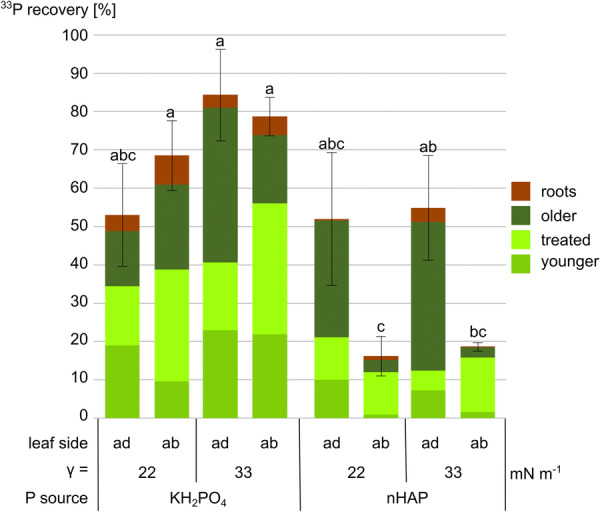
Recovery of ^33^P 7 days after foliar application of spiked KH_2_PO_4_ and nHAPs in greenhouse cultivated P deficient potato plants. ^33^P spiked KH_2_PO_4_ and nHAPs were applied to both leaf sides of the outermost leaflets of YFELs at the indicated surface tensions γ. The plot shows the recovery in different plant parts after 7 days as a percentage of the total applied ^33^P. The different plant parts are colour coded as indicated to the right hand side. ad.: adaxial treatments, ab.: abaxial treatment. *N* = 3, error bars show standard deviations for single fractions, letters indicate statistically different means by ANOVA, α = 0.05. [Color figure can be viewed at wileyonlinelibrary.com]

In a climate chamber experiment, ^33^P labelled KH_2_PO_4_ and nHAP were applied to YFELs of P− potato plants at γ = 33 mN m^−1^, and γ = 22 mN m^−1^, and the P recovery in different plant tissues was quantified after 7 d. The results are shown in Figure 5.

At γ = 33 mN m^−1^, the uptake of KH_2_PO_4_ was around 80%, regardless of the leaf polarity. Strikingly, 55% of ^33^P from nHAPs was taken up on the adaxial leaf side, while the uptake on the abaxial leaf side remained low at around 20% (Figure 5). This is irrespective of the fact that the stomatal density was 4 times lower on the adaxial leaf side than on the abaxial side (Figure 1f).

Lowering γ to 22 mN m^−1^ resulted in a slightly (not significant) reduced uptake of ^33^P from KH_2_PO_4_. It did not change the uptake efficiency for nHAPs compared to γ = 33 mN m^−1^. Generally, a reduction in γ did not appear to affect the translocation of applied P to other tissues.

In cases where > 50% uptake of P occurred, application to the adaxial leaf side resulted in stronger translocation of P to other tissues compared to application on the abaxial leaf side.

**Figure 6:**
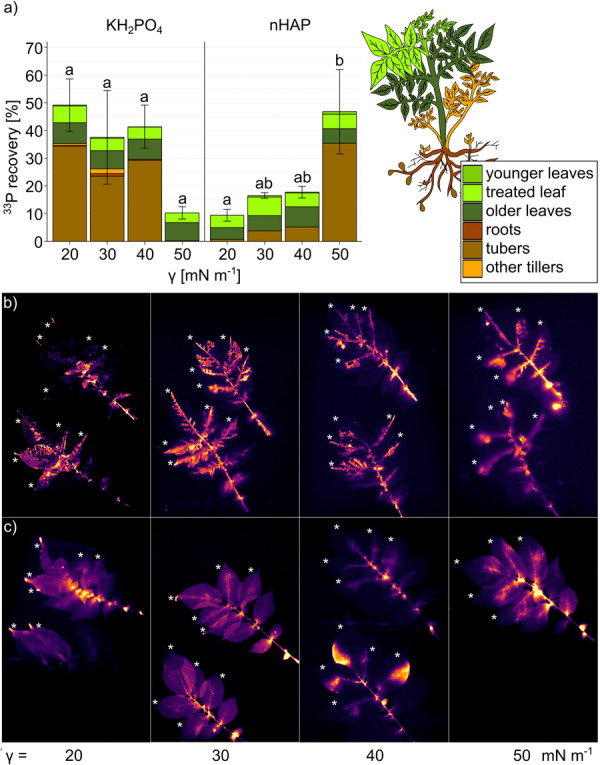
(a) Foliar P uptake efficiency in the field. Recovery of ^33^P from foliar fertiliser application on potato plants in the field at the late growth stage. The graph shows ^33^P recovery from ^33^P‐spiked KH_2_PO_4_ and nHAP (NP) into the indicated plant fractions after 7 days. The foliar solutions were applied as droplets at the indicated surface tensions. The graph in shows the average total uptake efficiency into treated potato plants, with the contribution of different plant fractions indicated in colours. Error bars show the SE for the mean total uptake. Letters indicate groups of significant differences for KH_2_PO_4_treatments and nHAP treatments separately (*N *= 4, ANOVA, α = 0.005). (b and c) ^33^P distribution in YFELs of potato plants grown in the field. Images show the ^33^P distribution for two replicate plants 7 days after treatment with either ^33^P‐spiked nHAP (b) or ^33^P‐spiked KH_2_PO_4_ (c) at the indicated surface tension. Treated leaflets are marked with an asterisk (*). [Color figure can be viewed at wileyonlinelibrary.com]

In order to translate the experiments obtained under controlled conditions into a real‐world scenario, a field trial was performed. ^33^P spiked KH_2_PO_4_ and nHAPs were applied at four different surface tensions to YFELs of potato plants in the tuber bulking phase (August 2024), and plants were harvested after 7 days. Temperature, precipitation, and leaf wetness data can be found in Supporting Information: Figure S7. Within the week, several precipitation events, including rain and morning dew, rewetted the plants. Due to large variations in the uptake efficiencies, no systematic differences between treatments were found (Figure 6a), although interesting trends were observed:

^33^P uptake from nHAP reached, 47% at ɣ = 50 mN m^−1^ (Figure 6a). Lowering the surface tension to γ = 20 mN m^−1^ resulted in a marked reduction in uptake efficiencies ( ~ 9%). At surface tensions of γ ≤ 40 mN m^‐1^, only a minor fraction of ^33^Pi was transported to the tubers, while this fraction dominated uptake at 50 mN m^−1^ (Supporting Information: Figure S8).

For ^33^P_i_, the uptake efficiency was highest at γ = 20 mN m^−1^ (49%) and decreased when γ was raised (Figure 6a). Most ^33^P_i_ was found in the tubers and older plant tissues, while no ^33^P_i_ seemed to be translocated to younger leaves (Figures 6b and S8). At γ = 50 mN m^−1^, no ^33^P_i_ was found in the tubers and uptake was only about 10%, indicating limited uptake and no translocation of ^33^P_i_.

Generally, translocation of ^33^P from the treated leaf to younger parts of the same shoot, as well as into roots and other tillers, contributed little to the total of ^33^P taken up. This was observed irrespective of the form ^33^P was applied in Figures 6b and S8.

The five outermost leaflets of YFELs of potato plants were treated alongside plants from the quantitative uptake experiment in the field. YFELs were harvested after 7 d, and the distribution of ^33^P in the treated leaf was imaged with phosphor‐screens after rinsing the leaf surface (Figure 6b,c).

When ^33^P was supplied as nHAP, a strong signal followed the veins, becoming weaker towards the periphery of the leaflets (Figure 6b). In contrast, when supplied as ^33^Pi, the signal distributed more evenly on the whole leaflet, while accumulating in distinct spots along the main vein (Figure 6c). In all treatments, strong signals were observed in non‐treated leaflets of the treated leaf, as well as in the leaf petiole leading to the tiller (Figure 6b,c), indicating translocation of ^33^P within the plant. Notably, these distribution patterns differed between ^33^P signal from nHAPs versus Pi (compare non‐treated leaflets in Figure 6b *vs.* 6c).

At low γ, tips of treated leaflets showed increased ^33^P signal, indicating loss by dripping from the leaf. At γ ≥ 40 mN m^−1^, the positions of droplet deposition were still visible after 7 d in most leaflets, indicating limited droplet spreading/water film development (Figure 6b,c).

Signal from nHAPs (Figure 6b), covered a larger leaf area at low γ, and accessed smaller leaf structures (i.e. smaller veins) on the surface. At higher γ, gradually less of the leaf surface appeared to be covered by the suspension, and the signal was confined to the location of droplet deposition and large veins. For ^33^P_i_, a similar but less pronounced distribution pattern can be observed only at γ = 50 mN m^−1^.

It is worth noting that all leaves were re‐wetted by dew formation at night as well as rain events several times during the field experiment (Supporting Information: Figure S7).

## 4 Discussion

### 4.1 Foliar NHAP Uptake Can Be Efficient

**Figure 7:**
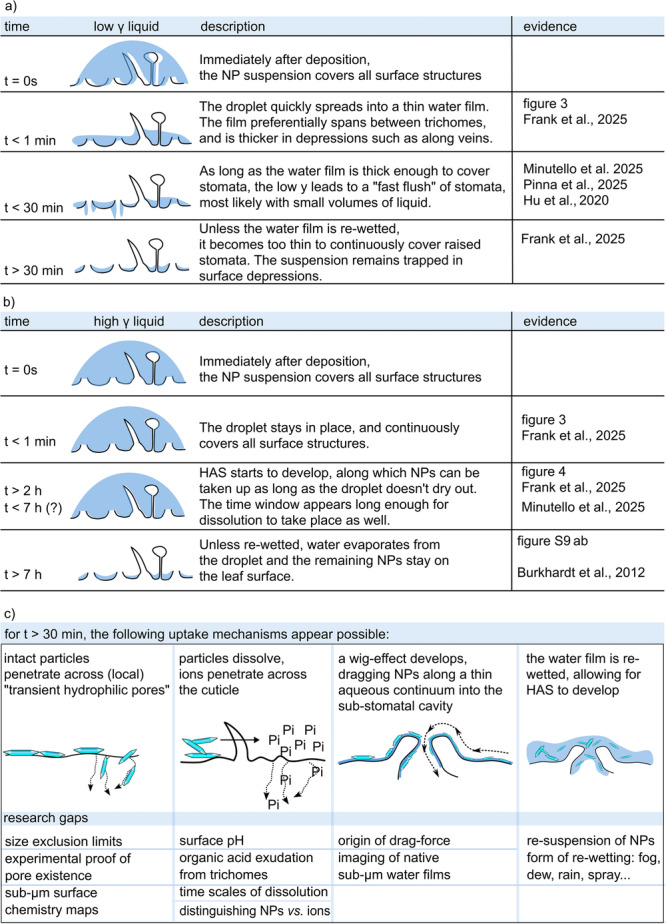
A theoretical model describing adaxial liquid‐surface interaction on potato leaves. (a) describes the development of a water film on a potato leaf at low ɣ over time, and (b) for high ɣ. (c) describes the potential entrance pathways for intact nHAPs and released Pi, and the research gaps which need to be addressed in order to resolve uncertainties. [Color figure can be viewed at wileyonlinelibrary.com]

It was encouraging to observe that the foliar uptake efficiency of P from nHAP reached 55% in the climate chamber and 47% in the field trial within 7 d after application (Figures 6 and 7). These values are far above the global uptake efficiency of P fertilisers within the first year after application to agricultural soils, which typically ranges between 10% and 25% (Syers et al. 2008; MacDonald et al. 2011). The foliar uptake efficiency of conventional P_i_ fertiliser was generally higher and reached up to 84% in the climate chamber and 49% in the field (Figures 6 and 7). However, when applied in agronomically relevant concentrations, P_i_ induced severe leaf scorching, whereas nHAP did not (Supporting Information: Figure S9). This underlines the potential of nHAP as a promising source of foliar P fertilisation in concentrations relevant for real world application. Future studies will need to investigate to which extent this can translate into improved fertilisation strategies.

The field trial data showed that Pi released from nHAPs was physiologically accessible to the plants, as it exhibited the same rapid translocation to tubers as the dissolved Pi treatment (Figure 6a). LA‐ICP‐MS mapping (Figure 4) and phosphor screen imaging (Figure 6b,c) further confirmed that nHAPs partially dissolved either on the leaf surface or after penetrating the leaf. The physiological functionality of similar particles has recently been demonstrated in barley (Minutello et al. 2026). In potatoes, however, demonstrating such functionality is more difficult because chlorophyll a fluorescence methods are poorly suited to this species due to the rapid remobilisation of P out of the treated leaves (Tougaard et al. 2023). In our greenhouse experiments, all attempts to reverse P deficiency failed ‐ even P‐deprived plants resupplied with triple doses of P in the hydroponic solution showed no signs of physiological recovery (data not shown). This outcome emphasises the exceptionally high P demand of potato plants described in the literature (Koch et al. 2019; Thornton 2020), likely because any available P is immediately directed toward shoot and/or tuber growth rather than restoring P functionality in various leaf positions.

Strikingly, the PUE of Pi and nHAPs differed in relation to leaf polarity (Figure 5) and liquid surface tension (Figure 6a). In the climate chamber experiment, nHAP uptake was ~2.5x more efficient on the adaxial leaf side than on the abaxial side (Figure 5). In contrast, Pi uptake was not affected by leaf polarity. Therefore, treatments were only applied to the adaxial side in the field trial. While nHAP uptake increased with higher ɣ, Pi uptake increased with lower ɣ (Figure 6a). Both taken together supported the hypothesis that nHAPs and Pi would require different entrance pathways and would thus be affected in different ways by leaf polarity and ɣ.

While we show that leaf properties vary with leaf age, our quantitative observations are based on one‐time treatments of YFELs. However, in a real‐world cropping system, fertiliser sprays would target both young and old tissues simultaneously, and the canopy properties including the ratio between leaf ages would vary across plant growth stages. Therefore, potential spraying outcomes and yield implications should be addressed in further trials.

### 4.2 Foliar NHAP Entry Pathways Relate to Uptake Efficiencies

Based on scientific literature, we must assume that the nHAPs used in this study (needle shaped, median size of 33.6 nm x 5.3 nm) would penetrate the leaf surface preferentially across stomata (Husted et al. 2023). Although available evidence is limited to a few plant species, the reported cuticular size exclusion limits range between 1 and 5 nm (Eichert and Goldbach 2008; Eichert et al. 2008). The entry of nHAPs across stomata must require an aqueous continuum, stretching from the leaf surface across the stomatal guard cell into the sub‐stomatal cavity, referred to as the hydraulic activation of stomata (HAS) (Burkhardt et al. 2012). A previous study has shown that HAS develops in open, adaxial stomata at ɣ = 70 mN m^‐1^, within 5 h after droplet deposition ((Frank et al. 2025), reproduced in Figure 3c). We verified this finding by choosing an intermediate surface tension of ɣ = 50 mN m^‐1^ and CLSM imaging after 2 h. This reassured us in the finding that HAS in adaxial potato leaves does neither require low ɣ (Schönherr and Bukovac 1972), nor several weeks to develop (Grantz et al. 2018), which had been the previous state of knowledge (Frank et al. 2025). Next, we utilised LA‐ICP‐MS to track Ce doped nHAP_Ce_ uptake across stomata 5 h after treatment (Figure 4). However, this was challenging: Ce signal originating from dialysed nHAPs entered the leaf tissue across stomata, but also across the leaf cuticle, into gT, and along cell walls at bases of ngT, irrespective of ɣ. Based on *in vitro* and *in vivo* dissolution experiments (Minutello et al. 2026), we had to assume that nHAPs had been intact when delivered to the leaf surface. Consequently, the following mechanisms might explain our findings, depicted in the working model (Figure 7c): (1) intact nHAPs were capable of penetrating the leaf cuticle across ‘transient hydrophilic pores’ (concept described in (Fernández et al. 2016)) which would be larger than expected, (2) nHAPs (partially) dissolved on the leaf surface, followed by Pi and Ce uptake across the hydrated cuticle, along with uptake of intact nHAPs via hydraulically activated stomata, or (3), a combination of (1) and (2). Option (1) would require cuticular pore sizes of > 5 nm, which has, to our knowledge, not been reported in literature. Potato leaf cross‐sections imaged by TEM did not reveal any visible pores (Figure 2a), but their existence cannot be excluded in the hydrated/native state. Previous studies observed that NPs with large aspect ratios displayed the ability to cross leaf cuticles of tomato leaves, but plausible explanations for this finding are still missing (Zhang et al. 2023). Overall, it appears more likely that nHAPs partially dissolved on the leaf surface. Organic acids secreted by gT in chickpea has been shown to dissolve foliar dust deposits, followed by uptake of Pi (Gross et al. 2021; Lokshin et al. 2026). Surprisingly, the effect was stronger in cultivars with higher trichome density and under P starvation (Golan et al. 2025). A similar phenomenon has not been observed for potato plants, but evidence of gT secretion in the *Solanum* family is increasing (Balcke et al. 2017; Koul et al. 2021). Option (2) is also supported by the phosphor‐screen data we show in Figure 7b,c: If nHAPs were fully dissolved, the ^33^P signal distribution would match with the signal of Pi (Figure 6b *vs.* 6c). However, this is only partly the case, indicating a mix of intact and dissolved particles present in the leaf tissue. Methods for proving the coexistence of dissolved and intact particles within the same sample would require two distinct signals that are specific to the intact particle and its dissociated components, which requires further method development (discussed in (Pinna and Husted 2025). Overall, we conclude that the majority of nHAPs would still rely on HAS for foliar penetration.

Therefore, it is important to understand the conditions under which HAS occurs. For better understanding, the processes discussed here are illustrated in a model (Figure 7a,b). In the field trial, we found that the PUE from nHAPs on the adaxial leaf side was most efficient at high ɣ. This fits well with the observation that HAS develops adaxially at high and intermediate ɣ (Figure 3c,g,h), but not equally at low ɣ (Frank et al. 2025). This is related to the raised topography of stomata (Figures 2b and 4a,b) and the fact that low ɣ water films rapidly spread out into thin layers (Supporting Information: Figure S5), failing to fully cover the stomatal opening over an extended period (Frank et al. 2025). However, a small quantity of nHAPs might enter *via* stomata at low ɣ as long as the water film still covers the pore immediately after droplet deposition (illustrated in Figure 7a), a process referred to before as ‘rapid flushing of stomatal pores’ in plants with sunken stomata (Hu et al. 2020).

While we fail to explain the high adaxial uptake efficiency of nHAPs in the climate chamber (Figure 5), the contrast between efficient adaxial *versus* inefficient abaxial uptake might also be understood because of stomatal shape and/or intra‐stomatal surface chemistry differences. Although the lower leaf side had 2.5x more trichomes than the upper side (Supporting Information: Figure S3, potential source of organic acids for nHAP dissolution), an about 40x higher stomatal density (Figure 2f, potential nHAP uptake pathway), and a similar wax/carbohydrate ratio (Supporting Information: Figure S4), the PUE from nHAPs was still higher on the upper leaf side (Figure 5). Therefore, we investigated how the ‘volcano shape’ of stomata on the adaxial leaf side might affect HAS development as opposed to the ‘tube shaped’ stomata on the abaxial leaf surface. We found that abaxial stomata were not penetrated by water, although fully covered by the water film, 3.5 h after droplet deposition (Figure 3f). Instead, gas from within the sub‐stomatal cavity seemed to push out into the overlying liquid, just as postulated in 1972 by (Schönherr and Bukovac 1972). Although this might be a single‐time point observation, we never observed this for adaxial stomata. Further lowering ɣ, as done in the climate chamber experiment, would result in a poorer coverage of stomata (Frank et al. 2025). (Fernández et al. 2024) has shown that the leaf surface chemistry can vary drastically within a nm scale, especially around surface structures. Therefore, it seems possible that our observation is connected to different guard cell and intra‐stomatal surface chemistries based on leaf polarity.

### 4.3 Pi Penetrates Potato Leaves Across the Cuticle

We utilised vanadate (Vi) as a proxy for foliar uptake of Pi, due to the high P background in plants. Previous work including speciation analysis has shown that Vi and Pi behave similarly in plant tissues, as long as Vi is used in concentrations below the toxicity level (Arsic et al. 2020). The same study showed that Pi was capable of entering barley leaves across fibre cells and bundle sheath extensions along veins, which has been related to transient hydrophilic pore formation by swelling of pectin enriched cell walls under high RH (Arsic et al. 2020; Arsic et al. 2022). Here, we find adaxial penetration of Vi across all surface structures of YFELs in potato (Supporting Information: Figure S6), indicating the presence of hydrophilic pores in the leaf of a dicot. LA‐ICP‐MS data showed surface penetration both in presence and in absence of a surfactant (Supporting Information: Figure S6). However, it is well established that foliar absorption of solutes generally improves with better leaf wetting, i.e. a larger contact area between liquid and leaf cuticle (Stevens 2006; Peirce et al. 2019). Therefore, low ɣ is often recommended, which might come with the side effect of cuticle disintegration or even disruption, especially when organosilicone based surfactants are used (Zhang et al. 2020; Zhang et al. 2021). We observe the expected tendency in our field trial: Foliar Pi uptake improves with lower ɣ. It further underlines that foliar Pi uptake, in contrast to nHAP uptake, does not equally depend on stomata. Consequently, the uptake efficiency does not differ between leaf sides (Figure 5). It is worth noting, however, that low ɣ can also have detrimental effects, such as loss due to dripping (visible as strong signal on tips of treated leaflets in Figure 6c) and adverse environmental effects of the surfactant ((Chen et al. 2018; Li et al. 2019).

### 4.4 NHAPs Partly Translocate as Intact Particles

It was interesting to see that Ce signal from nHAP_Ce_ accumulated within the vasculature of the treated leaflet (Figure 4b) as well as the connected petiole (Figure 4a,b) within 5 h after foliar exposure. As discussed above, further method development is required to verify that nHAPs were intact at this stage. However, the ^33^P radioisotope maps shown in Figure 7b,c seem to support the interpretation that at least a fraction of the ^33^Pi is transported within the vasculature as intact nHAPs, because the signal detected in non‐treated leaflets differs markedly between the Pi and the nHAP treatments. Signals from nHAPs followed the vasculature more strictly, even in leaflets that had not been exposed to the treatment (Figure 7b). In contrast, Pi spread out over the whole blade of leaflets more evenly (Figure 7c), which is also reflected in the fact that Vi distributed all over the leaf tissue, with no preference to accumulate around the leaf vasculature (Supporting Information: Figure S6). In a recent study, we observed a similar accumulation of a water soluble contrast agent (iohexol) around the vasculature of potato young potato plants (Frank et al. 2025). While a variety of studies seems to indicate that nano materials may translocate within the phloem, potential phloem loading mechanisms of NPs remain poorly understood, especially with respect to size exclusion limits of intact cell walls, plasmodesmata and pore‐plasmodesmatal units (Pinna and Husted 2025). For instance, it remains unclear whether positive xylem pressure (Schenk et al. 2021) could induce a counterflow which would result in long distance transport within the plant. However, once dissolved, nHAPs will yield Pi, which is highly phloem mobile and is remobilized to sink tissues within hours (Tougaard et al. 2023). In barley, nHAPs dissolved within the apoplast in regions of foliar penetration over the course of 1‐7 days, gradually releasing Pi (Minutello et al. 2026). Therefore, *in planta* mobility of nHAPs might not be of outmost importance with respect to efficient foliar P delivery. Once taken up, Pi from nHAPs shows a similar distribution within the plant as Pi, both in the climate chamber in young plants (Figure 5) and in the field in plants in the tuber bulking phase (Figure 6a and Supporting Information: Figure S8).

### 4.5 Perspectives

In this work, we show that foliar P fertilisation with nHAPs can be an efficient way to accommodate the high P demand of potato plants while avoiding Pi immobilisation in soils. We conclude that nHAPs and Pi follow different entry pathways into potato leaves and therefore show differences with regards to the optimal formulations and application strategies. In order to fully understand how nHAPs cross foliar surface barriers as opposed to free ions, it will first be necessary to elucidate the size exclusion limits of cuticular pores, and how these might be located in relation to specific leaf surface structures. Secondly, it would require collecting spatial information that is specific to intact NPs, either by utilising quantum dots (QDs) in confocal microscopy, or by exploiting the potential of LA‐single‐particle‐ICP‐time‐of‐flight‐MS (LA‐spICP‐TOF‐MS). Re‐wetting by high relative humidity, dew and rainfall, as well as repeated opening and closure of stomata, adds another layer of complexity, which remains un‐resolved but may have major implications for foliar NP uptake. Ultimately, future field trials will need to translate the mechanistic insights from the present study into applicable fertilisation strategies and yield responses. We believe that understanding the behaviour of foliar NP suspensions on individual leaf surfaces, potentially altered by a history of aging under nutrient deficiency or herbivory, is the way forward for more responsible fertilisation practices.

## 1 Conflicts of Interest

The authors declare no conflicts of interest.

## Supporting information

Supporting Data:

Supporting Video:

## Data Availability

All raw data are available on the Electronic Research Data Archive (ERDA) of the University of Copenhagen: https://sid.erda.dk/cgi-sid/ls.py?share_id=d4s2vLK0O1.
