# Supplementary material for: Distinct Foliar Uptake Pathways for Phosphorus and Nano‐Hydroxyapatite in Potato Revealed By Synchrotron μCT and ³³P Imaging
Source: Plant Cell Environ. 2026 Apr 22;49(8):5563–79. doi: 10.1111/pce.70558 (PMC13353727; doi:10.1111/pce.70558)
Supplement: Supplementary file 1 — Supporting Data: [file PCE-49-5563-s002.docx]

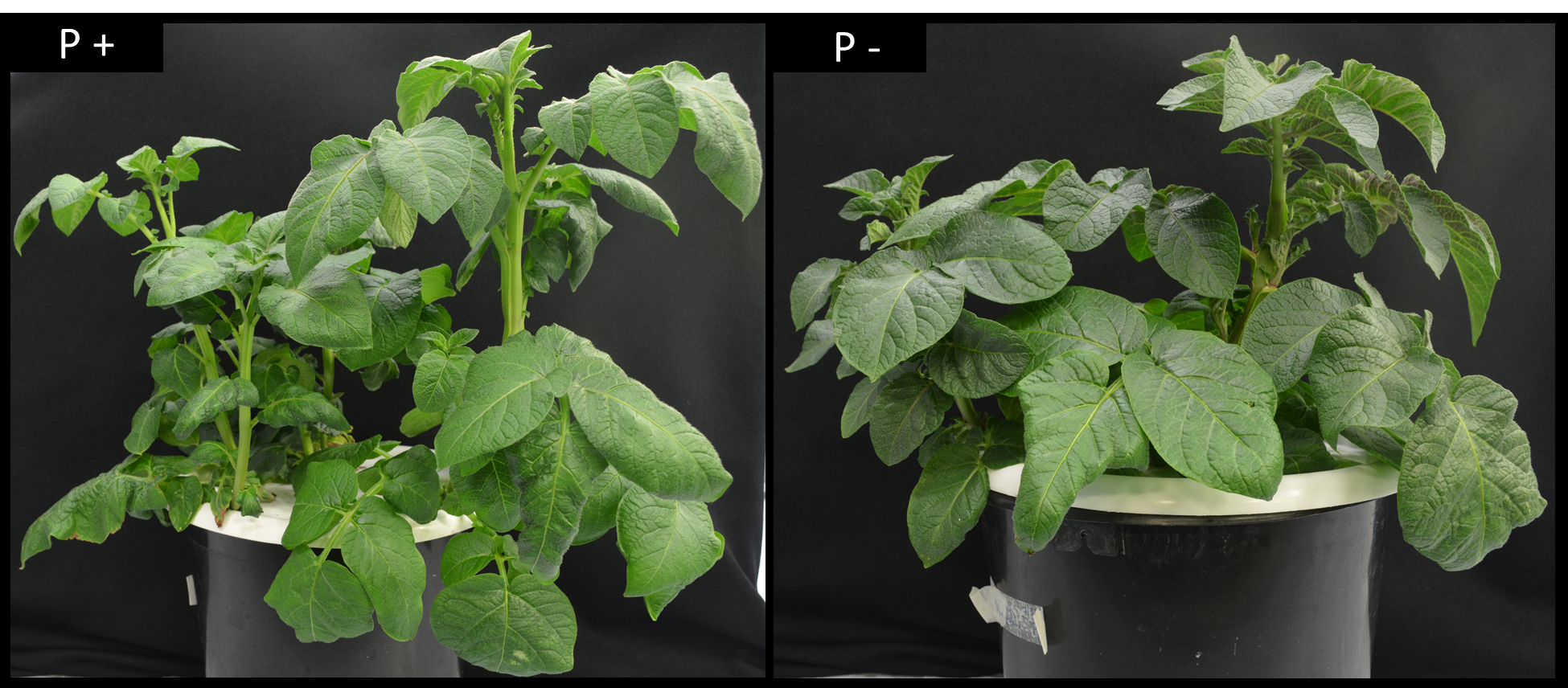


**Figure S1:** Potato plants in hydroponic culture. P sufficient (left) plants had extended internodes, light green leaves, and did not display signs of anthocyanosis, while P deficient plants (right) displayed typical P deficiency symptoms, including stunted growth with short internodes and darker leaves with anthocyanosis.


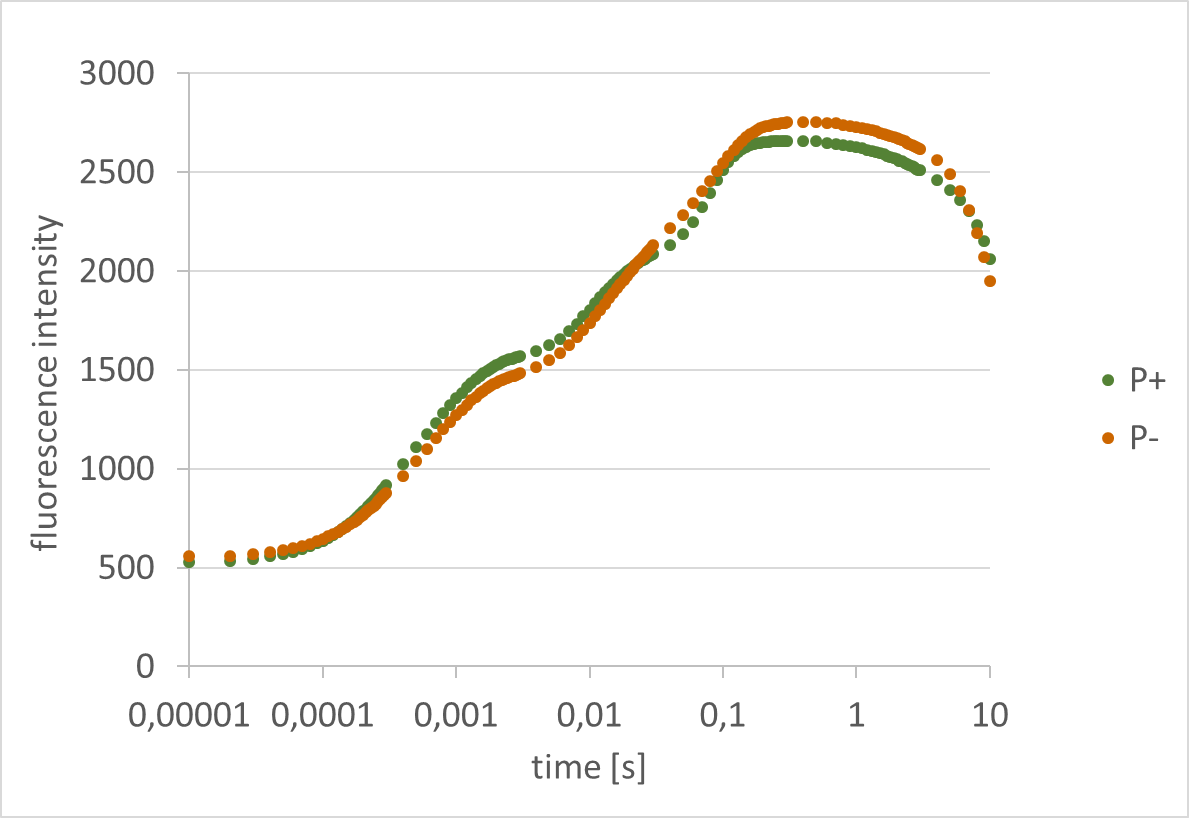


**Figure S2:** OJIP transients of P sufficient (**P+**, n = 5) and P deficient (**P-**, n = 6) potato plants grown in hydroponic culture. YFELs of P- plants displayed a flattened I-step in the OJIP transient, reflecting fast stroma acidification under high light intensities, as well as a faster decline of the transient after its maximum compared to P+ treatments.

**
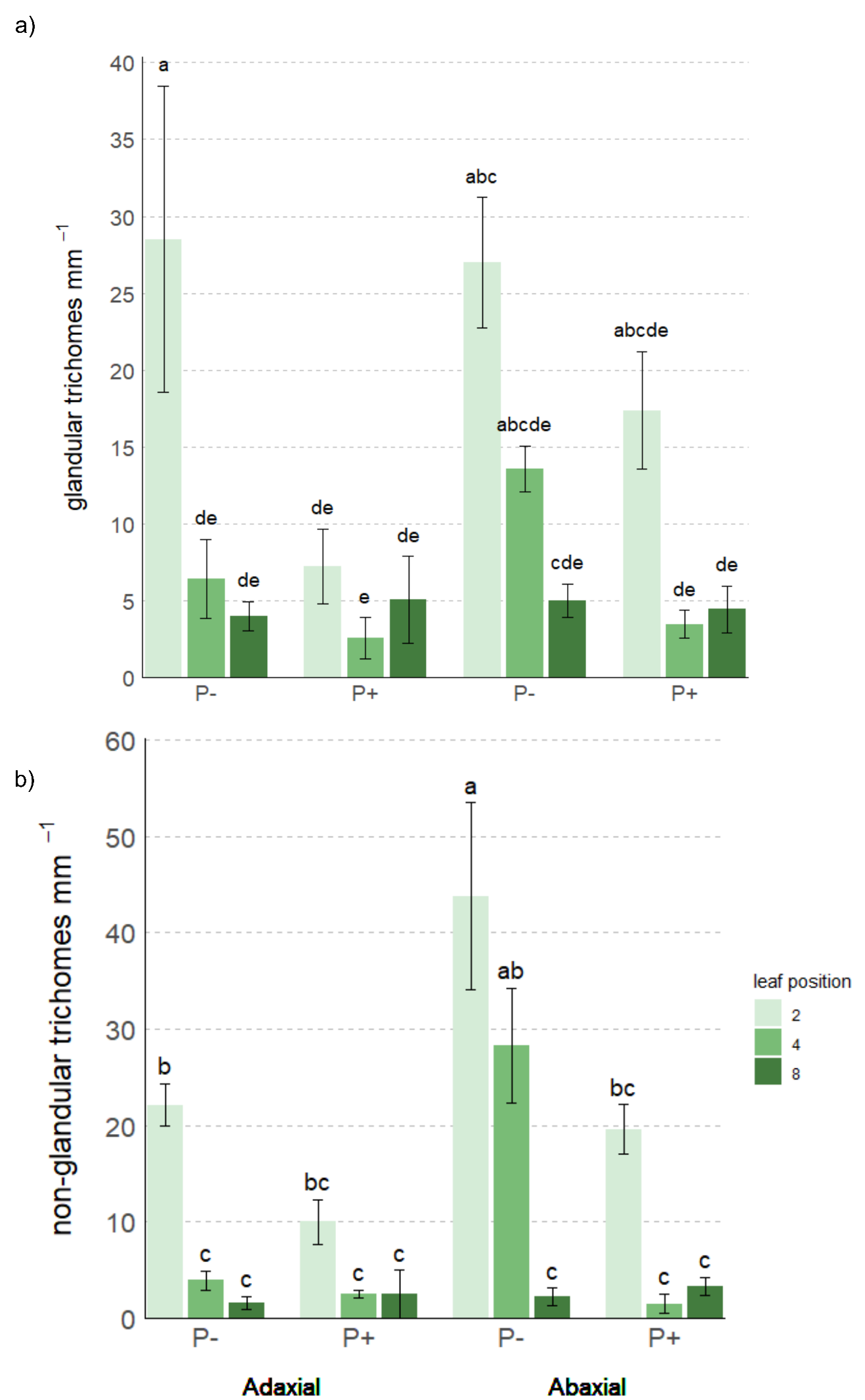
**

**Figure S3:** Trichome density on leaves of potato plants. Density of glandular (**a**) and non-glandular (**b**) trichomes on adaxial and abaxial leaf sides of leaf positions 2 (youngest leaf), 4 (YFEL), and 8 (oldest leaf) of P deficient (P-) and P sufficient (P+) potato plants. Trichomes were counted in 4-7 randomly chosen areas on the leaf on SEM micrographs. Error bars indicate standard deviations, and letters indicate groups of significance based on an α $\leq$ 0.05 significance level (Šidák test).

**
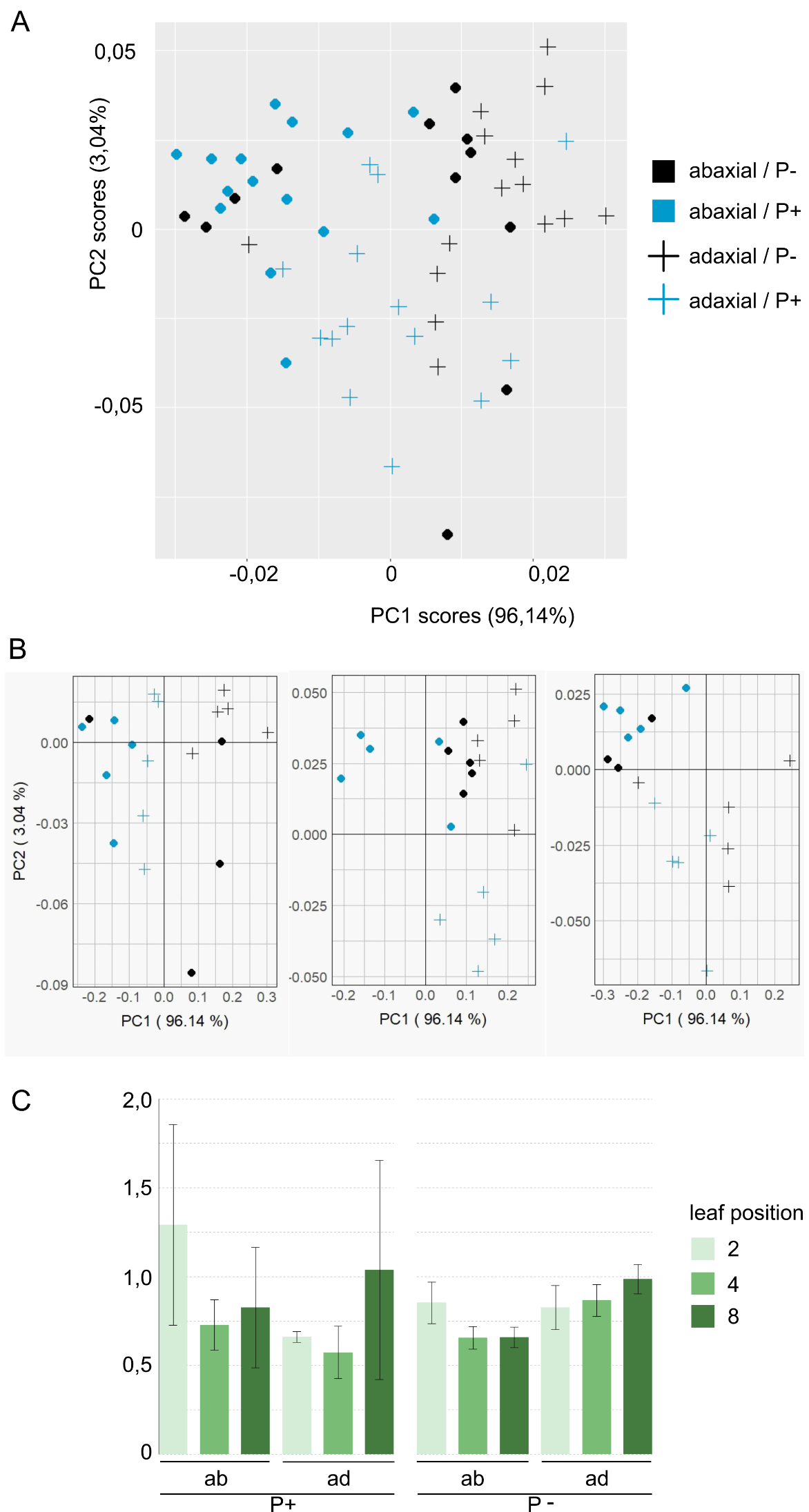
**

**Figure S4:** Potato leaf surface chemistry analysis. A and B: Principal component analysis for explaining wax (PC1) and carbohydrate (PC2) composition of the leaf surface as a result of adaxial and abaxial side of P deficient (P-) and P sufficient (P+) potato plants in leaf positions 2, 4, and 8. C: Wax to carbohydrate ratio on adaxial and abaxial side of leaves in position 2, 4, and 8 of P deficient (P-) and P sufficient (P+) potato plants grown in hydroponics. Error bars show the sd.


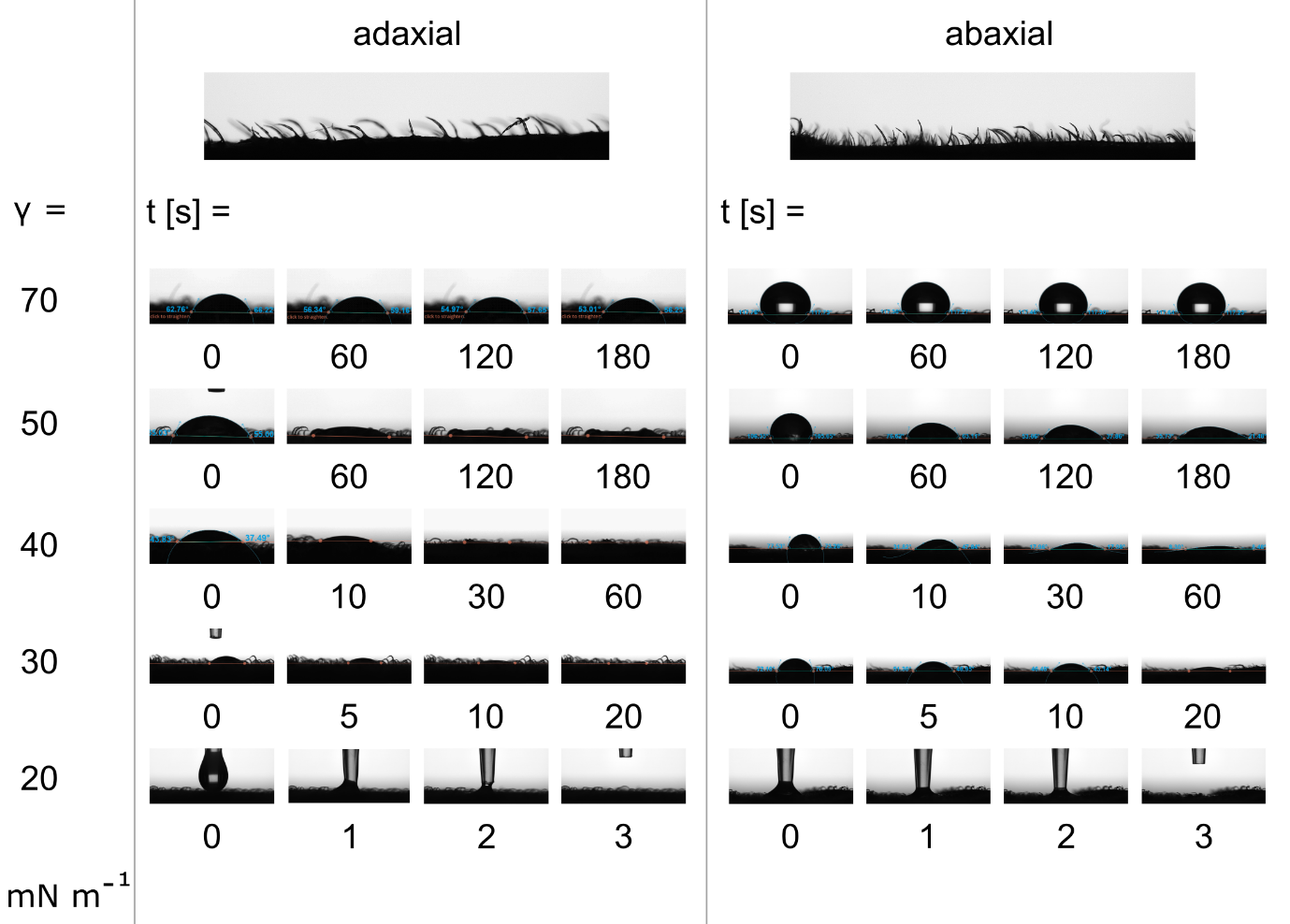


**Figure S5**: Droplet spreading on potato leaves. Optical tensiometry reveals the spreading dynamics of droplets on the adaxial (left) and the abaxial (right) leaf side at the indicated surface tensions and time points. For additional quantitative information, see supplementary figure 8.


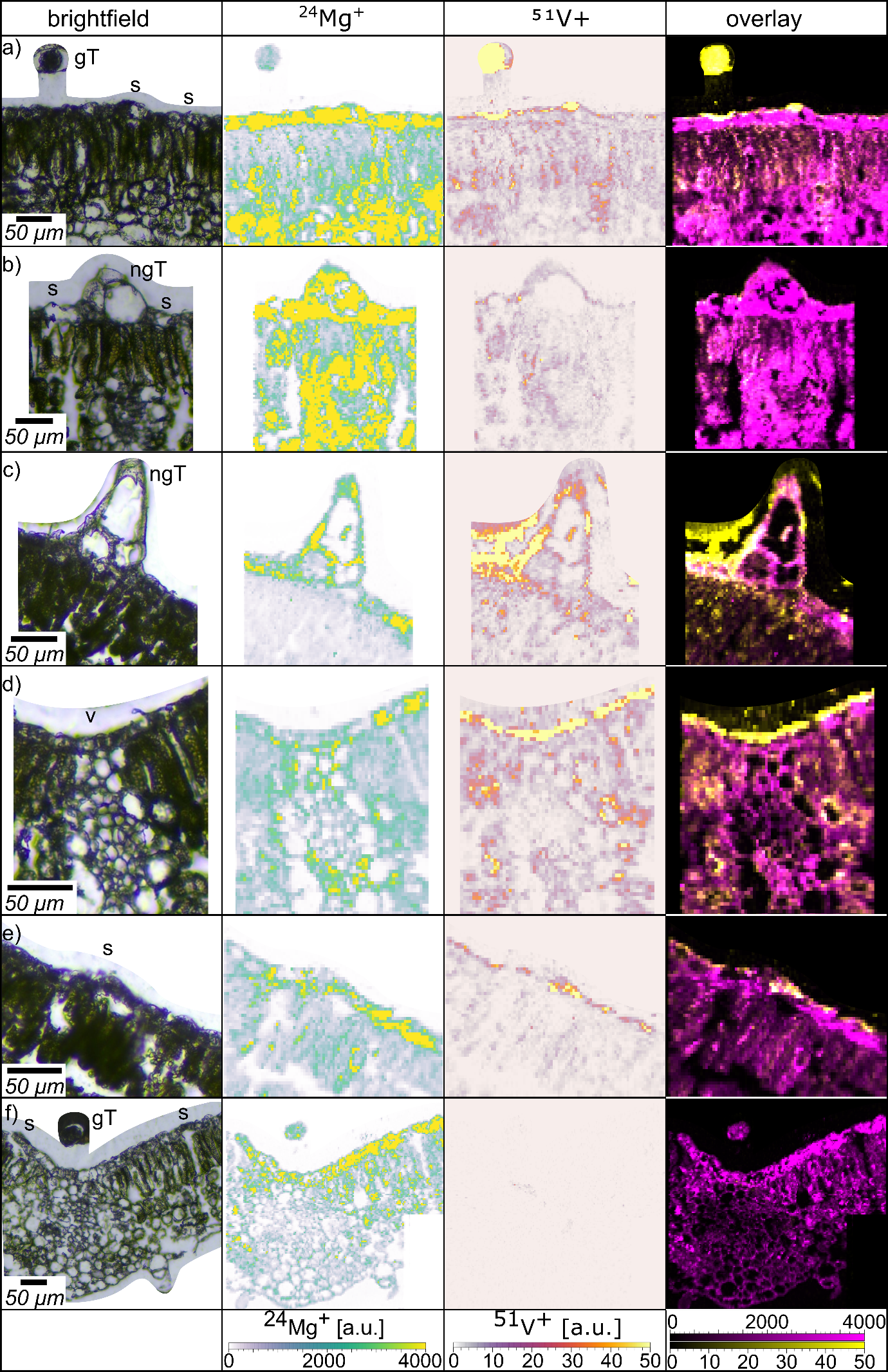


**Figure S6:** Light microscopy and LA-ICP-MS based elemental maps of vanadate treated YFEL cross-sections harvested after 24h. **a**) and **b**): VO_4_^2-^ at γ =70 mN m^-1^. **c**) to **e**): VO_4_^2-^ at γ =24 mN m^-1^. **f**): Control, surfactant only. D and F are sections through leaf veins. s: stoma, ngT: non-glandular trichome, gT: glandular trichome. Note that the units of the color scales correspond to ion counts by the ICP-MS, but that the images are non-quantitative by nature of the analysis technique.


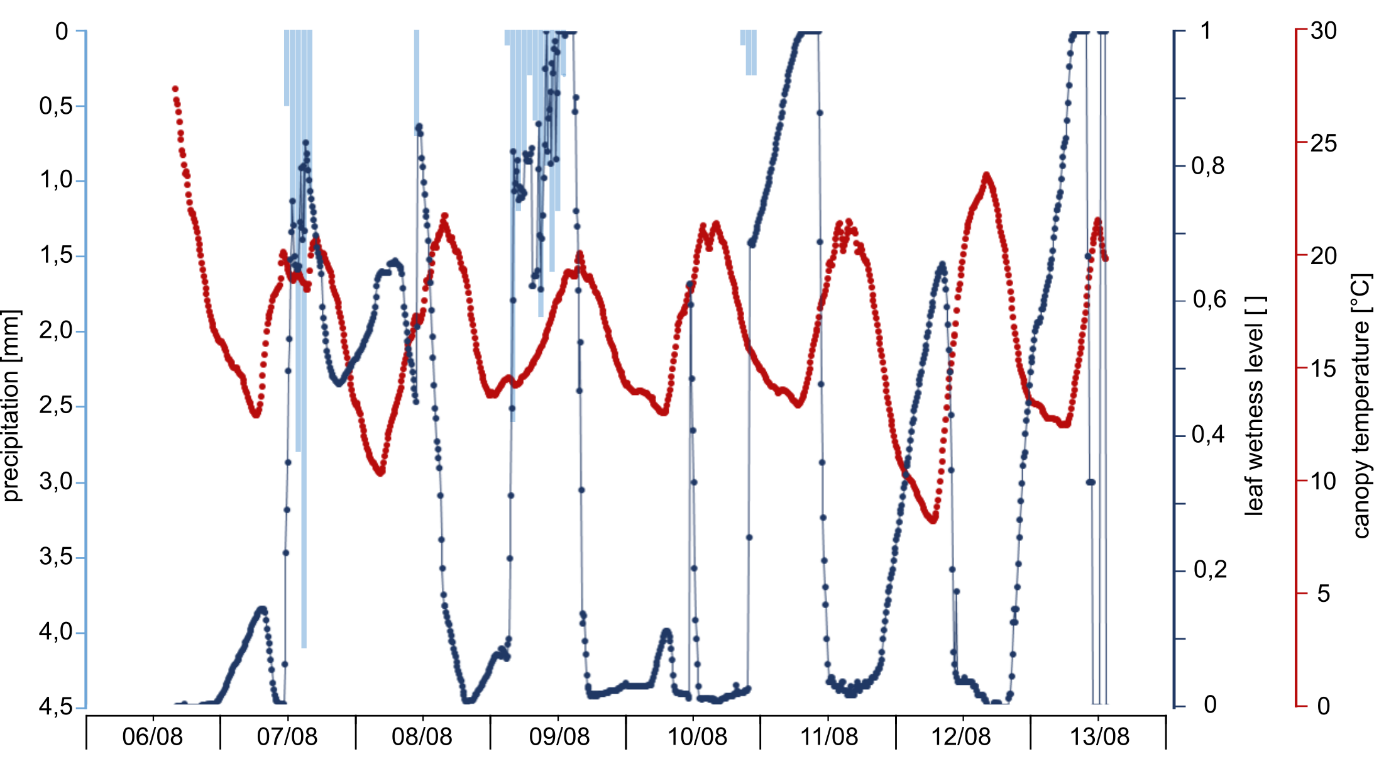


**Figure S7**: Weather data during the field trial in Jutland from 6^th^ -13^th^ of August 2024. Light blue bars: Precipitation data from DMI. Dark blue curve: normalized leaf wetness index obtained from a leaf wetness sensor mounted in the canopy on the height of YFELs. Red curve: Temperature measured in the canopy on the height of YFELs.


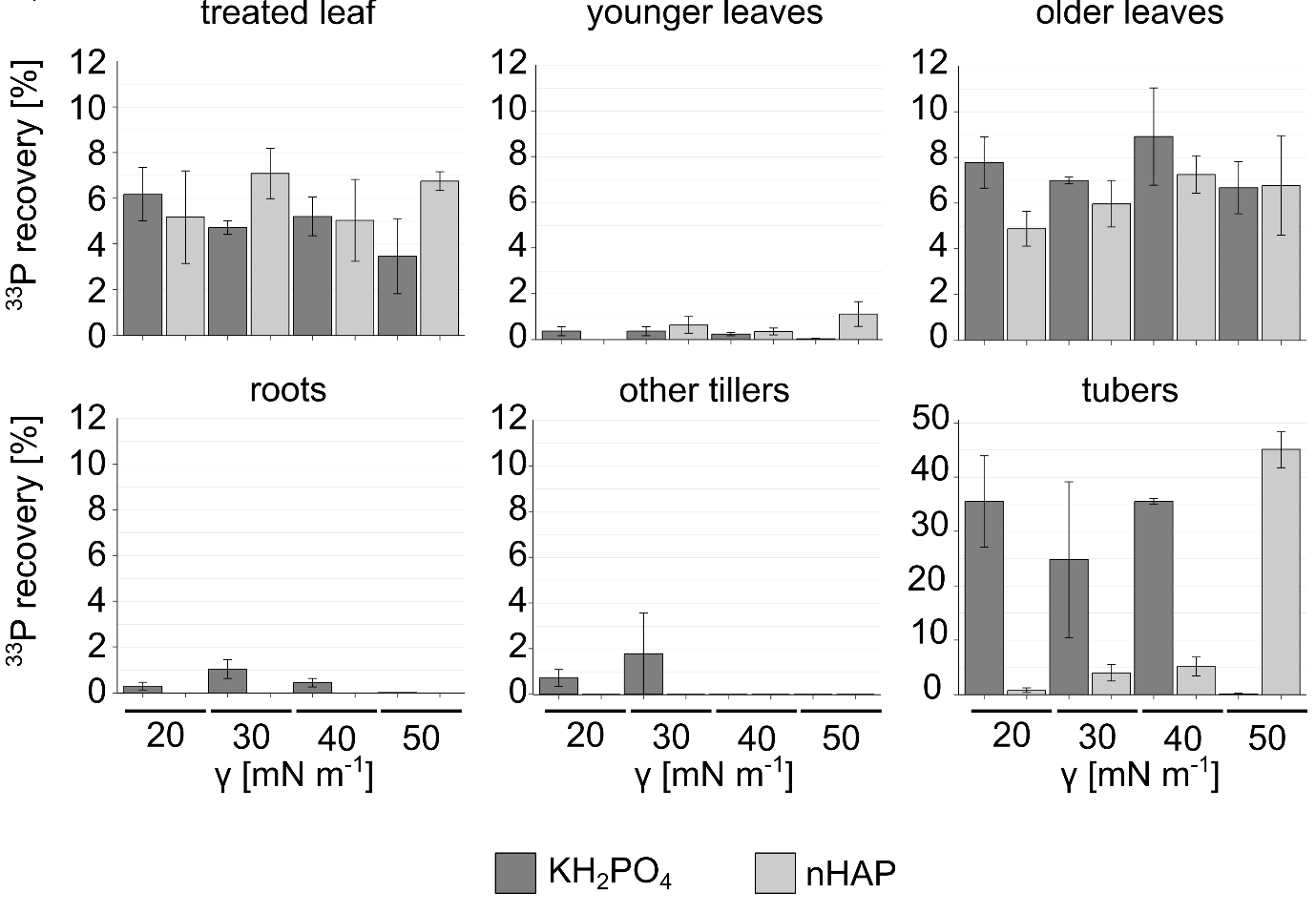


**Figure S8:** Foliar P uptake efficiency in the field. Recovery of ^33^P from foliar fertilizer application on potato plants in the field at the late growth stage. The graph shows ^33^P recovery from ^33^P-spiked KH_2_PO_4_ (KP) and nHAP* (NP) into the indicated plant fractions after 7 days. The foliar solutions were applied as droplets at the indicated surface tensions. Percentage of ^33^P from the treatment taken up into the different plant fractions. Error bars show the sd, N = 4. No statistical differences are indicated.

**
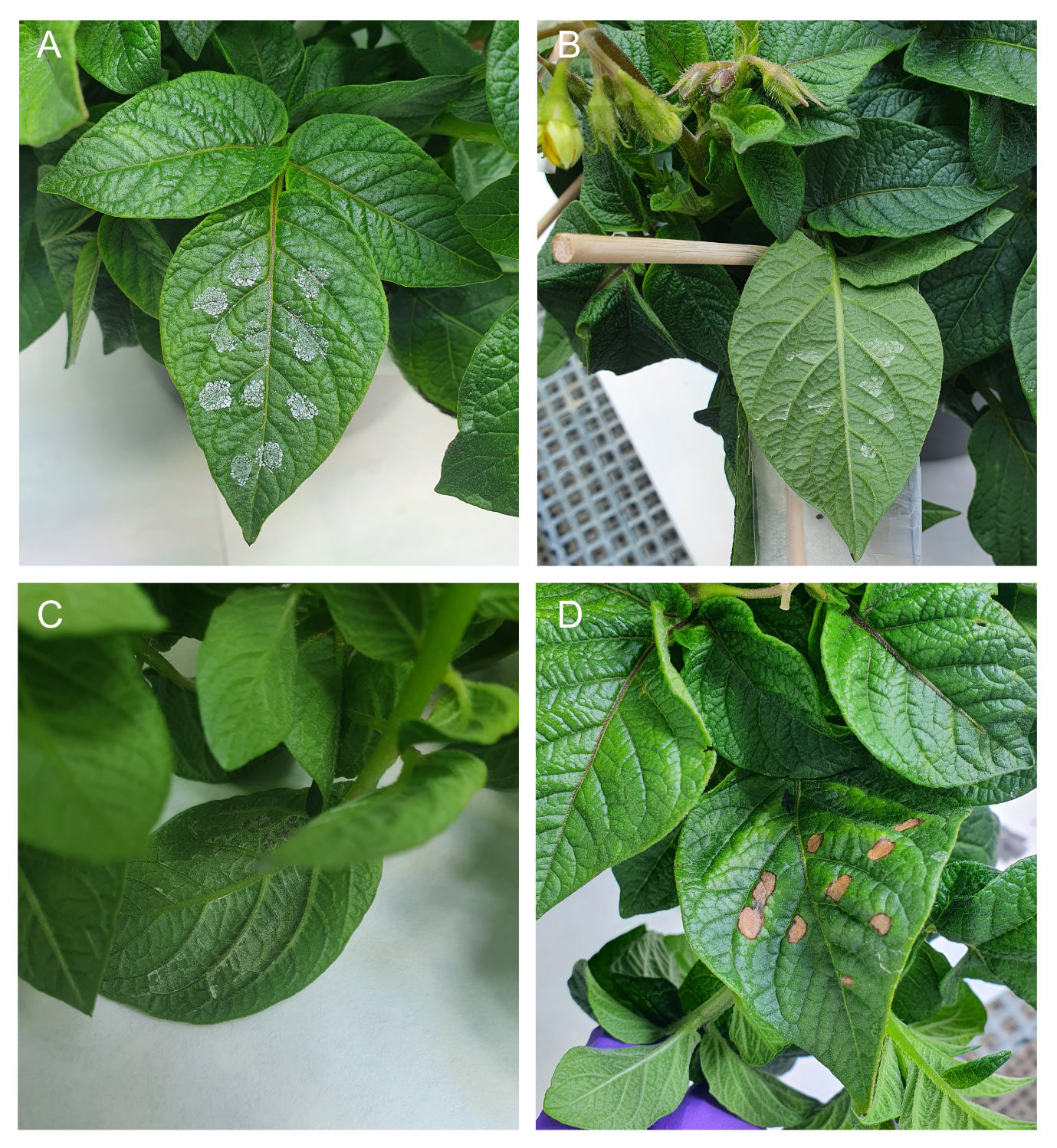
**

**Figure S9:** Leaf scorching test. Images show YFELs of P- potato plants 1 week after application of foliar droplets of nHAP* (A-C) and KH_2_PO_4_* (D) at a P concentration of 5 mg ml^-1^ in the climate chamber. A, B, D: 44 mN m^-1^. C: 22 mN m^-1^. A and D: adaxial application. B and C: abaxial application.
